# A long intergenic non-coding RNA regulates nuclear localization of DNA methyl transferase-1

**DOI:** 10.1016/j.isci.2021.102273

**Published:** 2021-03-05

**Authors:** Rhian Jones, Susanne Wijesinghe, Claire Wilson, John Halsall, Triantafillos Liloglou, Aditi Kanhere

**Affiliations:** 1 School of Biosciences, University of Birmingham, Edgbaston, Birmingham, UK; 2 Institute of Inflammation and Ageing, University of Birmingham, Edgbaston, Birmingham, UK; 3 Institute of Systems, Molecular and Integrative Biology, University of Liverpool, Liverpool, UK; 4 Institute of Genomic Sciences, University of Birmingham, Edgbaston, Birmingham, UK

**Keywords:** Molecular Biology, Cell Biology

## Abstract

DNA methyl transferase-1 or DNMT1 maintains DNA methylation in the genome and is important for regulating gene expression in cells. Aberrant changes in DNMT1 activity and DNA methylation are commonly observed in cancers and many other diseases. Recently, a number of long intergenic non-protein-coding RNAs or lincRNAs have been shown to play a role in regulating DNMT1 activity. *CCDC26* is a nuclear lincRNA that is frequently mutated in cancers and is a hotbed for disease-associated single nucleotide changes. However, the functional mechanism of *CCDC26* is not understood. Here, we show that this lincRNA is concentrated on the nuclear periphery. Strikingly, in the absence of *CCDC26* lincRNA, DNMT1 is mis-located in the cytoplasm, and the genomic DNA is significantly hypomethylated. This is accompanied by double-stranded DNA breaks and increased cell death. These results point to a previously unrecognized mechanism of lincRNA-mediated subcellular localization of DNMT1 and regulation of DNA methylation.

## Highlights

• LincRNA CCDC26 influences cellular localization of the enzyme DNMT1
• In the absence of CCDC26, the genomic DNA is significantly hypomethylated
• Removal of CCDC26 leads to double-stranded DNA breaks and increased cell death

## Graphical abstract

**Figure:**
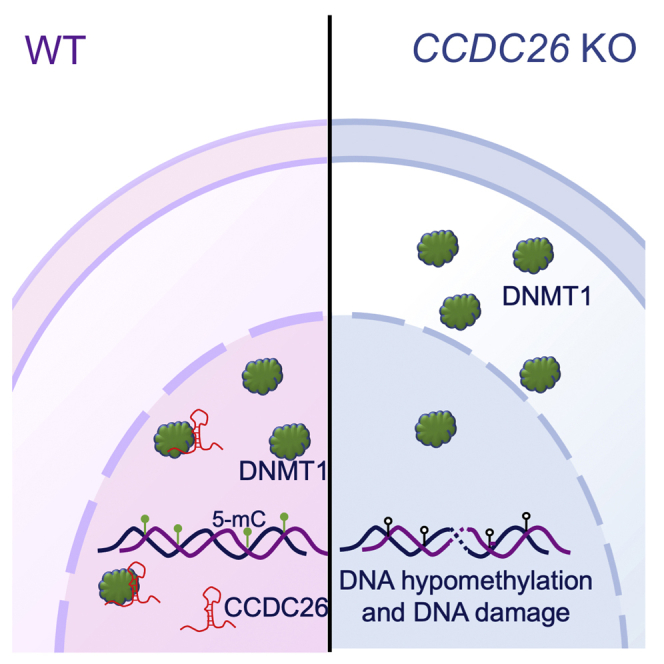

## Introduction

In the mammalian genome, DNA is often methylated at cytosines in CpG dinucleotides. DNA methylation is one of the important epigenetic modifications needed for transcriptional regulation of genes (Jaenisch and Bird, 2003). This modification plays a crucial role in many vital cellular processes such as heterochromatin formation, X-chromosomal inactivation, and genomic stability (Csankovszki et al., 2001; Nan et al., 1998; Watt and Molloy, 1988). Unsurprisingly, aberrant DNA methylation is implicated in many diseases and developmental defects (Baets et al., 2015; Daskalos et al., 2009; Eden et al., 2003; Gaudet et al., 2003; Heller et al., 2016; Klein et al., 2013; Morgan et al., 2018; Pakneshan et al., 2004; Schmelz et al., 2005; Stirzaker et al., 1997; Wen et al., 2018; Yu et al., 2013). Regulation of DNA methylation is therefore crucial throughout mammalian existence.

In mammals, DNMT3a, DNMT3b, and DNMT1 are DNA methyltransferases that are responsible for establishing and maintaining genomic methylation in cells (Lyko, 2018). DNMT3a and DNMT3b are primarily involved in establishing *de novo* DNA methylation patterns in the genome. In the very early stages of development, DNA methylation is completely eradicated in primordial germ cells, resulting in an epigenetically “blank canvas.” The DNMT3s then restore DNA methylation in a non-CpG-specific and ubiquitous manner (Doherty et al., 2002; Otani et al., 2009; Rasmussen and Helin, 2016). In contrast, DNMT1 plays a more predominant role in maintaining post-replicative methylation patterns, by preferentially binding hemi-methylated DNA, and methylating the newly synthesized daughter strand (Lyko, 2018). At late S-phase of the cell cycle, DNMT1 is targeted to replication foci, a process dependent on an additional ubiquitin-like protein, with PHD and RING finger domains 1 (UHRF1) (Berkyurek et al., 2014; Bostick et al., 2007; Bronner et al., 2019). Evidence suggests that during the replication process, DNMT1 also interacts with histone-modifying enzymes such as histone deacetylase HDAC2 as well as histone methyltransferases, EZH2 and G9a (Esteve et al., 2006; Rountree et al., 2000; Wang et al., 2017).

Aberrant DNA methylation is suspected to play a role in many cancers, e.g., hepatocellular carcinoma, glioblastoma, breast cancer, squamous cell lung cancer, thyroid cancer, and leukemia, (Daskalos et al., 2009; Eden et al., 2003; Gaudet et al., 2003; Heller et al., 2016; Morgan et al., 2018; Pakneshan et al., 2004; Schmelz et al., 2005; Stirzaker et al., 1997; Wen et al., 2018; Yu et al., 2013), and DNMT mutations are also the cause of developmental diseases such as hereditary sensory and autonomic neuropathy type 1E (HSAN1E) (Baets et al., 2015; Klein et al., 2013).

In somatic cells, DNMT1 is the most abundant and most active methyl transferase. It is a predominantly nuclear protein with an N-terminal nuclear localization signal (NLS) stretching between 177 and 205 amino acid residues (Alvarez-Ponce et al., 2018). The N-terminus of DNMT1 also contains domains required for its interaction with partner proteins, including DMAP1, HP1, G9a, and PCNA (Esteve et al., 2006; Fuks et al., 2003; Iida et al., 2002; Rountree et al., 2000). The central region of DNMT1 is needed for its targeting to replication foci (Leonhardt et al., 1992), whereas the C-terminus comprises the catalytic domain required for methyl-transferase activity (Song et al., 2012).

A number of studies have shown that DNA methylation is regulated by non-protein-coding RNAs or ncRNAs (Berghoff et al., 2013; Imamura et al., 2004; Jeffery and Nakielny, 2004; Yu et al., 2008). Also, DNMT1 function is often influenced by its interactions with ncRNAs. Long ncRNAs or lncRNAs such as *KCNQ1OT1* (Mohammad et al., 2010), *Dali* (Chalei et al., 2014), *lincRNA-p21* (Bao et al., 2015), *PARTICLE* (O'Leary et al., 2017), *ecCEBP* (Di Ruscio et al., 2013), *DACOR1* (Somasundaram et al., 2018), and *HOXA11-AS1* (Guo et al., 2019) are shown to interact with DNMT1 and modulate its activity. Here we report an interaction between DNMT1 and a long intergenic non-coding RNA (lincRNA), *CCDC26*. LincRNA *CCDC26* is transcribed from a 328-kilobase gene on chromosome 8, from 8q24.21 locus neighboring the proto-oncogene c-MYC. The 8q24 locus is a hotbed for disease-associated mutations including cancer-associated SNPs and copy-number alterations (Wilson and Kanhere, 2021). It is of specific interest in acute myeloid leukemia (AML) because of the high-frequency occurrence of AML-associated mutations and variants in *CCDC26* gene (Duployez et al., 2018; Izadifard et al., 2018; Kuhn et al., 2012; Radtke et al., 2009). These observations suggest that *CCDC26* might play an important role in driving cancer progression. Previous studies show that *CCDC26* might be involved in regulating apoptosis and differentiation in myeloid cells (Yin et al., 2006; Hirano et al., 2015). However, the functional mechanism of *CCDC26* remains elusive.

In this study, we aimed to understand the function of *CCDC26* and its role in cancer. Here, we show that *CCDC26* interacts with DNMT1 and is predominantly localized on the nuclear periphery. In the absence of *CCDC26*, DNMT1 is mis-localized in the cytoplasm, leading to DNA hypomethylation and apoptosis similar to that observed on inhibition of DNMT1 in myeloid leukemia cells. As a result, we observe genome-wide changes in gene expression. LincRNA-mediated DNMT1 mis-localization has not been previously reported and has significant implications to DNA methylation regulation, as well as cancer and RNA biology.

## Results

### LincRNA *CCDC26* is a myeloid-specific RNA expressed from second TSS

We first sought to understand the gene structure and expression pattern of *CCDC26*. Previous investigations (Hirano et al., 2015; Yin et al., 2006) on various cell types showed that *CCDC26* is highly expressed in myeloid leukemias. We further examined this by measuring *CCDC26* levels in a number of additional leukemia and non-leukemia lines (Figure S1A). In agreement with previous results, we observed that *CCDC26* is highly expressed in myeloid cells and is present at much lower levels in other cell types. We also observed that among myeloid cells the highest level of expression is in chronic myeloid cells, K562 (Figure S1A).

**Figure 1:**
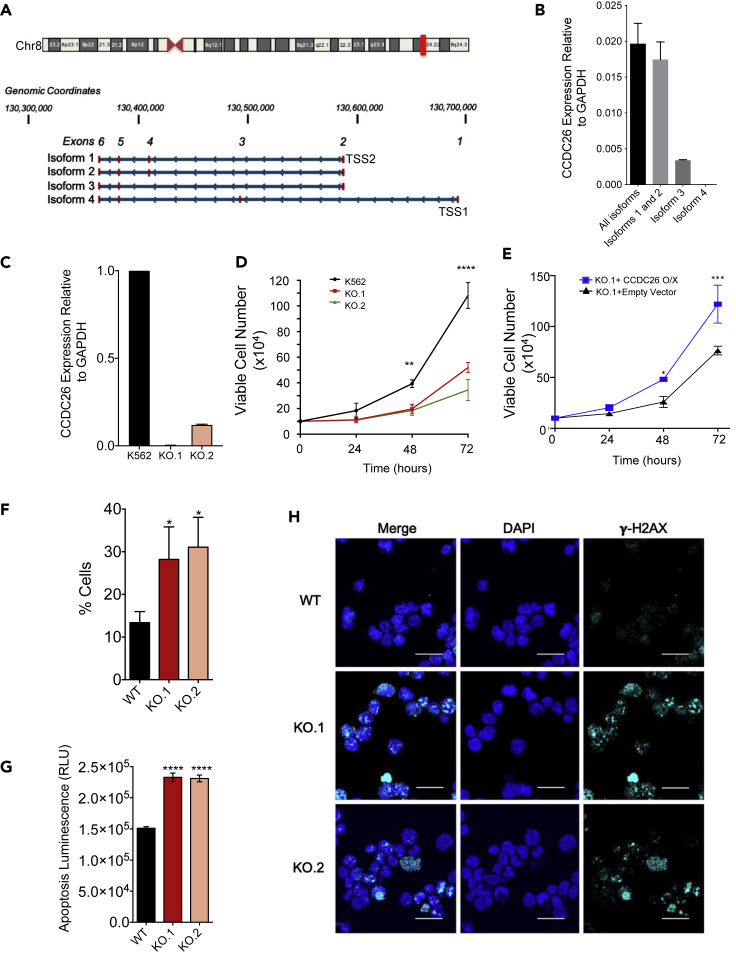
LincRNA *CCDC26* knockout results in slower growth and increased apoptosis and DNA damage in K562 cells (A) A schematic diagram illustrating the four currently known isoforms of *CCDC26*, adapted from the UCSC Genome Browser view of the *CCDC26* locus (NCBI RefSeq tracks, GRCh37/hg19 assembly. Ideogram for chromosome 8 is shown at the top. The red baron ideogram indicates *CCDC26* position. *CCDC26* isoforms are transcribed from one of two transcription start sites (TSS-1 and TSS-2) located on Chr8q24 locus. (B) A plot showing the RNA levels of different *CCDC26* isoforms, relative to GAPDH, in K562 cells, measured using qRT-PCR. Error bars represent the mean ± standard deviation. (C) A plot showing *expression* level of *CCDC26* RNA relative to GAPDH in WT K562, KO.1, and KO.2 cells measured using qRT-PCR. *CCDC26* levels were significantly reduced in both knockouts. Error bars represent the mean ± standard deviation. (D) Growth curve for WT K562, KO.1, and KO.2 cells showing slower growth in the latter cell lines. Values represent the mean ± standard deviation. ∗p < 0.05; ∗∗p < 0.005; ∗∗∗p < 0.001; ∗∗∗∗p < 0.0001 (unpaired, two-tailed t test, n = 3). (E) Growth curve for KO.1 expressing *CCDC26* from exogeneous plasmid vector and KO.1 expressing empty vector. Values represent the mean ± standard deviation. ∗p < 0.05; ∗∗p < 0.005; ∗∗∗p < 0.001; ∗∗∗∗p < 0.0001 (unpaired, two-tailed t test, n = 3). (F) A plot showing the percentage of apoptotic WT, KO.1, and KO.2 cells in the sub-G1 phase of the cell cycle according to propidium iodide FACS analysis. Values represent the mean ± standard deviation. ∗p < 0.05, ∗∗p < 0.005; ∗∗∗p < 0.001; ∗∗∗∗p < 0.0001 (unpaired, two-tailed t test, n = 3). (G) The ApoTox-Glo assay allows for detection of apoptosis in cells. The Caspase-Glo 3/7 reagent results in cell lysis, leading to caspase cleavage and the release of the luciferase substrate amino-luciferin, causing the luciferase reaction to emit light, indicating apoptosis. Luminescence was detected and measured for WT and *CCDC26* KO cells to determine the level of apoptosis in cells. Values represent the mean ± standard deviation. ∗p < 0.05; ∗∗p < 0.005; ∗∗∗p < 0.001; ∗∗∗∗p < 0.0001 (unpaired, two-tailed t test, n = 5). (H) Confocal images demonstrating the results of anti-**γ**-H2AX immunofluorescence. WT, KO.1, and KO.2 cells were stained with DAPI nuclear stain (blue) and anti-**γ**-H2AX antibody (cyan). Increased numbers of **γ**-H2AX foci are present in the KO cells. Scale bar, 25 um.

According to current gene annotations, altogether there are four isoforms of *CCDC26* that are transcribed from human chromosome 8 from two distinct transcription start sites, TSS1 and TSS2 (Figure 1A). These isoforms show alternative splicing patterns and contain combinations of six exons. Isoforms-1 (1666bp) and -2 (1649bp) are transcribed from an independent transcription start site (TSS2) and differ only by an additional 17 nucleotide sequence at the 3′ end of exon 4 in Isoform-1. Isoform-3 (1495bp), which is also transcribed from TSS2, lacks exon 4 completely. Isoform 4 (1718bp) is transcribed from TSS1, a start site that lies upstream of TSS2.

To understand which *CCDC26* isoforms are expressed in K562 cells, we carried out qRT-PCR measurements using isoform-specific primers (Figure 1B and Table S1). In these cells, the four isoforms of *CCDC26* are not uniformly expressed. Isoforms starting from TSS2 i.e. isoforms-1, -2, and -3 account for more than 95% of the total *CCDC26* transcripts in the cell. Among the three isoforms starting at TSS2, Isoform-1 and Isoform-2 alone make 80% of *CCDC26*, whereas Isoform-3 is detected at considerably lower levels, accounting for only ∼15% of *CCDC26* expression. On the other hand, Isoform-4, which is expressed from another transcription start site, TSS1, is barely detectable (Figure 1B).

### LincRNA *CCDC26* depletion leads to DNA damage and apoptosis

To further understand the role of *CCDC26* in myeloid cells, we carried out CRISPR-Cas9-mediated knockout (KO) of this lincRNA in K562 cells. Given that more than 99% of *CCDC26* is transcribed from TSS2, we simultaneously used two small guide RNAs to mutate TSS2 (Figure S1B). Following single-cell clonal expansion, we established two cell lines, KO.1 and KO.2, which exhibit ∼99% and ∼88% reduction in *CCDC26* levels, respectively (Figure 1C).

We first analyzed the effect of *CCDC26* knockout on cell growth. Interestingly, *CCDC26* depletion resulted in a significantly reduced rate of growth in both KO cell lines (Figure 1D). Cells were counted every 24 h across a 72 h period, and growth curves were subsequently plotted. Although wild-type (WT) cells double in number approximately every 24 h as previously reported (Murray et al., 1993), the number of KO cells only increase 2-fold approximately every 48 h (Figure 1D). A previous study (Hirano et al., 2015) also observed slower growth rate upon shRNA mediated knockdown of *CCDC26*. However, this effect was only observed under high serum conditions. The effect of *CCDC26* removal under normal growth conditions has not been reported before. Our results confirm that, even under normal conditions, *CCDC26* removal leads to reduced cell growth (Figure 1D). To further confirm this phenotype and to rule out the possibility that the slow growth in KO cells is a result of clonal selection after CRISPR knockouts, we compared the growth rates of KOs with clonal population transfected with CRISPR/Cas9 plasmid on its own without sgRNAs. However, this CRISPR control displayed similar growth rate as K562 cells (Figure S1C), supporting our observation that the slower growth rate observed in KOs is a likely consequence of *CCDC26* knockout and not a result of clonal isolation. We also reintroduced *CCDC26* in the knockouts using an exogenous plasmid vector. Overexpression of *CCDC26* in the knockouts increased cell growth rate as compared with knockouts that expressed an empty vector (Figures 1E and S1D), further supporting our observation that the cell growth changes are due to removal of *CCDC26*.

The slow growth observed in KO cells could be either because of changes in cell cycle or because of an increased rate of cell death. Previously, *CCDC26* has been implicated in apoptosis (Hirano et al., 2015; Yin et al., 2006); however, its effect on cell cycle has not been investigated. To understand the reason behind slow growth rate in KO cells, we subsequently analyzed cell-cycle progression in WT and KOs by incorporation of propidium iodide and subsequent FACS analysis. This method measures the number of DNA strands to determine cell cycle progression. We did not observe any significant changes in any of the key cell-cycle stages (Figures S1E–S1G). However, cell cycle analysis showed an increased population of *CCDC26* KO cells in the sub-G1 state, which is indicative of apoptosis (Figure 1F). Cell cytotoxicity and apoptosis assays that measure caspase levels also confirmed that in comparison to WT, *CCDC26* KO cells were less viable and more apoptotic (Figure 1G). The process of apoptosis is often linked to DNA damage. Hence, we explored the possibility of increased DNA damage in *CCDC26* KO cells. We tested for increased presence of histone variant **γ**-H2AX (Figure 1H). Histone variant H2AX is key in the cellular response to DNA damage, as its C-terminal tail is rapidly phosphorylated at a Serine residue following the occurrence of a DNA double-strand break (DSB). Phosphorylated H2AX or **γ**-H2AX is used as a DSB marker and can be readily detected by anti- **γ**-H2AX antibody (Rogakou et al., 1998). A visible increase in **γ**-H2AX foci was observed in both *CCDC26* KO lines as compared with WT cells (Figure 1H and Tables S2 and S3). Together, these results indicated that removal of *CCDC26* results in DNA damage, apoptosis, and slow growth.

### Absence of *CCDC26* leads to DNMT1 mis-localization and DNA hypomethylation

We further sought to understand the mechanism behind *CCDC26*-mediated DNA damage. We first enquired if this RNA influences genomic DNA and chromatin in any other way. Many lncRNAs are involved in regulation of chromatin modifications (Di Ruscio et al., 2013; Mohammad et al., 2010; Pandey et al., 2008; Rinn et al., 2007). We speculated that *CCDC26* might also function by regulating changes in epigenetic modifications. In order to verify this, we tested global genomic levels of multiple common histone modifications such as H3K27me3, H3K27ac, H3K9me3, H3K9ac, and H4K16ac using immunoblotting (Figure S2). However, we did not see any significant changes in genomic levels of tested histone modifications in *CCDC26* KO cells. Using immunoblotting, we also tested levels of histone-modifying enzymes such as EZH2, G9a, and HDAC2 (Figure S2) that are involved in catalyzing these modifications. However, similar to histone modifications, levels of these catalytic proteins did not show any changes. Although this observation does not rule out possibility of site-specific variations in histone modifications, these results indicate that absence of *CCDC26* did not lead to any significant changes in overall levels of histone modifications or the enzymes that catalyze these histone modifications.

**Figure 2:**
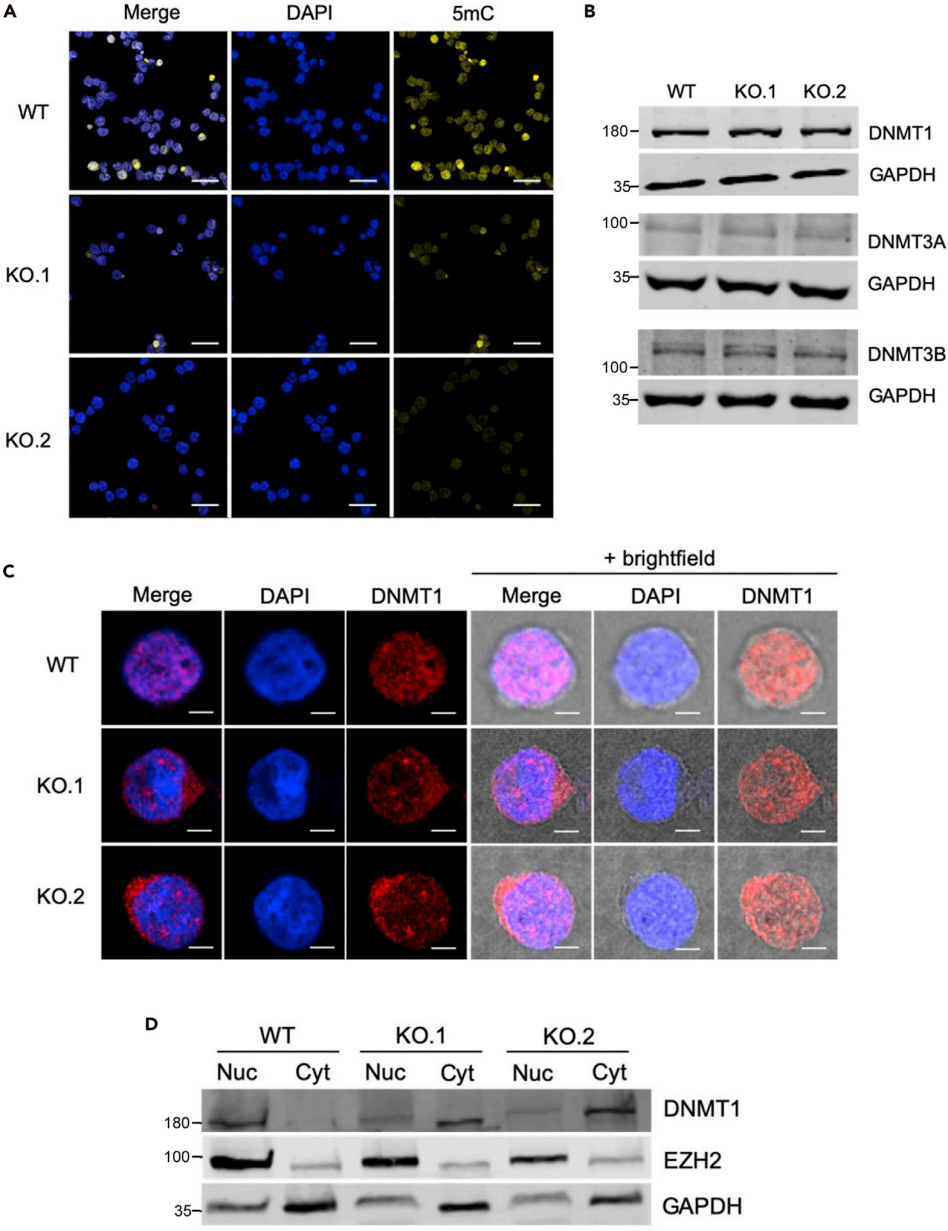
*CCDC26* knockout results in mis-localization of DNMT1 in the cytosol and global DNA hypomethylation (A) Confocal images demonstrating the results of anti-5mC immunofluorescence. WT, KO.1, and KO.2 cells were stained with DAPI nuclear stain (blue) and anti-5mC antibody (yellow). Reduced levels of 5mC fluorescence are observed in both KO cell lines. Scale bar, 50 um. (B) Total protein levels of DNMT1, DNMT3a, and DNMT3b measured relative to GAPDH by immunoblotting are unchanged in WT and *CCDC26* KO cells. (C) Confocal images demonstrating the results of anti-DNMT1 immunofluorescence. WT, KO.1, and KO.2 cells were stained with DAPI nuclear stain (blue) and anti-DNMT1 antibody (red). The outline of the cell membrane can be seen with the addition of the brightfield lens in the right-hand panels. DNMT1 is nuclear in WT cells and is largely cytosolic in the KO cells. Scale bar, 5 um. (D) Immunoblotting for DNMT1 on nuclear and cytosolic protein fractions shows a shift in the subcellular localization of DNMT1. DNMT1 is almost exclusively nuclear in the WT cells but appears both nuclear and cytosolic in *CCDC26* KO cells. EZH2 and GAPDH are used as nuclear and cytosolic markers, respectively (nuc = nuclear protein fraction; cyt = cytosolic protein fraction).

In addition to histone modifications, DNA methylation is an important epigenetic modification. Importantly, DNA methylation is also shown to be regulated by various lncRNAs (Bao et al., 2015; Chalei et al., 2014; Di Ruscio et al., 2013; Gao et al., 2020; Guo et al., 2019; Merry et al., 2015; Mohammad et al., 2010; O'Leary et al., 2017; Sun et al., 2016; Wang et al., 2015). To test if DNA methylation levels have changed in the KOs, we carried out immunofluorescence measurements using anti-5-methyl cytosine antibody (Figures 2A, S3A, Tables S2 and S3). Surprisingly, in KO lines, the 5-methyl cytosine signal was considerably weaker as compared with WT cells, indicating genomic DNA was hypomethylated (Figures 2A and S3A).

A potential explanation for hypomethylation of genomic DNA in *CCDC26* KO lines could be reduced levels of DNA methyltransferase proteins. However, immunoblotting in the *CCDC26* knockouts, showed no significant changes in the levels of the three DNA methyltransferase proteins, DNMT3a, DNMT3b, and DNMT1 (Figures 2B and S3B). Therefore, we speculated that DNA-binding capacity or the enzymatic activity of a DNA methyltransferase might have changed in the absence of *CCDC26*. To detect changes in the association of DNMTs with DNA, we performed anti-DNMT immunofluorescence. We observed that, in KOs, the subcellular localization of DNMT1 is predominantly cytosolic in contrast to WT where DNMT1 is, as expected, localized in the nucleus (Figure 2C). This was supported by immunoblotting measurements using anti-DNMT1 antibody on nuclear and cytosolic fractions of KOs as compared with WT (Figure 2D). However, mis-localization was not observed in case of DNMT3a and DNMT3b or other nuclear proteins such as HDAC2 (Figures S3C and S3D).

### Genes repressed by DNMT1 are upregulated in KO cells

**Figure 3:**
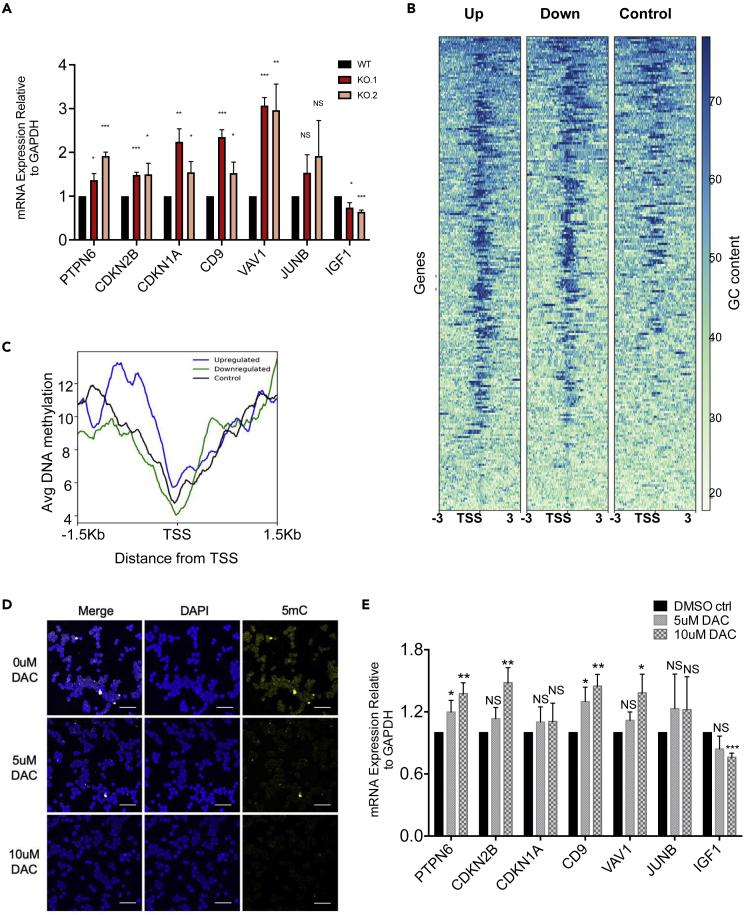
DNMT1 and methylation-regulated genes show expression changes in *CCDC26* KO cells (A) A plot showing expression levels of various genes whose expression has previously been shown to be impacted by DNMT1 depletion or DNA hypomethylation in myeloid leukemia. Levels are measured relative to GAPDH in WT K562, KO.1, and KO.2 cells by qRT-PCR. Values represent the mean ± standard deviation. ∗p < 0.05; ∗∗p < 0.01; ∗∗∗p < 0.001; NS = Not significant (unpaired, two-tailed *t* test). (B and C) (B) Heatmaps showing GC distribution around of Transcription Start Sites (TSS) of 2-fold downregulated and upregulated genes in KO as well as same number of randomly selected control set of genes. (C) Metagene plots of average enrichment of DNA methylation K562 cells at 2-fold downregulated genes (blue) as compared with upregulated genes (green) in JARID2 KO cells and random set of genes as control (black). The plots are centered on TSS of genes, and distance from TSS is indicated on the x axes. (D) Confocal images demonstrating the results of anti-5mC immunofluorescence on cells treated with either 0uM, 5uM, or 10uM DNMT1 inhibitor DAC. Cells were stained with DAPI nuclear stain (blue) and anti-5mC antibody (yellow). Reduced levels of 5mC fluorescence are observed in cells treated with 5uM and 10uM DAC. Scale bar, 50 um. (E) A plot showing expression levels of various genes (as in A) whose expression has previously been shown to be impacted by DNMT1 depletion or DNA hypomethylation in myeloid leukemia. Levels are measured relative to GAPDH in cells treated with 0-uM, 5-uM, and 10-uM DNMT1 inhibitor DAC. Values represent the mean ± standard deviation. ∗p < 0.05; ∗∗p < 0.01; ∗∗∗p < 0.001; NS = Not significant (unpaired, two-tailed *t* test).

We further hypothesized that if DNMT1 protein mis-localizes in the cytosol, then DNMT1 would be unable to carry out its primary function of methylating genomic DNA in the nucleus. Consequently, these cells should behave similarly to cells lacking DNMT1. To confirm this, we investigated expression levels of a selection of seven genes, previously shown to be significantly impacted by DNMT1 and methylation levels in myeloid cells (Figure 3A). These were protein tyrosine phosphatase non-receptor type 6 (*PTPN6*) (Li et al., 2017; Wang et al., 2017), cyclin-dependent kinase inhibitor 1A (*CDKN1A*) (Milutinovic et al., 2004; Schmelz et al., 2005), cyclin-dependent kinase inhibitor 2B (*CDKN2B*) (Herman et al., 1996; Yu et al., 2013), *CD9* (Kim et al., 2012), *VAV1* (Fernandez-Zapico et al., 2005; Ilan and Katzav, 2012), and *JUNB* (Fiskus et al., 2009; Yang et al., 2003), all of which have previously demonstrated upregulation in response to DNMT1 downregulation or DNA hypomethylation. We also measured levels of *IGF1*, as it has previously been reported that *IGF1* is repressed as a result of DNMT1 inhibition (Pastural et al., 2007). qRT-PCRs demonstrated that out of seven genes we tested, five (*PTPN6*, *CDKN1A*, *CDKN2B*, *CD9*, and *VAV1*) were significantly upregulated in both KO cell lines as reported in past studies on DNMT1 KD or inhibition. *JUNB*, albeit not significantly, also demonstrated upregulation. As expected, *IGF1* was significantly downregulated in both KO cell lines. This result demonstrates that gene expression changes in *CCDC26* KO cells are very similar to cells in which DNMT1 has been knocked down or inhibited. Presumably this is due to the unavailability of DNMT1 in the *CCDC26* KOs, given its predominantly cytoplasmic localization in these cells.

To confirm if these genes were upregulated as a result of DNA methylation changes at their promoters, we measured DNA methylation levels at the promoters of *PTPN6*, *VAV1*, *CD9*, and *CDKN1A* genes that were significantly upregulated in *CCDC26* KOs as compared with WT. DNA methylation levels were measured using bisulfite conversion and followed by a pyrosequencing assay. We observed that, with the exception of *CDKN1A*, promoter methylation was reduced at the tested genes in both KOs (Tables S4 and S5 and Figure S4A).

In order to verify this at the genomic level, using RNA-seq we measured genome-wide changes in gene expression. RNA-seq analysis showed that, in total, 287 genes showed more than 2-fold changes in RNA levels in both KO lines. Among these, 146 were upregulated and 141 were downregulated. It was previously shown that DNA methylation of CpG rich promoters leads to transcriptional repression (Kass et al., 1997; Li et al., 1993; Siegfried et al., 1999; Venolia and Gartler, 1983; Walsh et al., 1998). If gene expression changes in *CCDC26* KO are due to mis-localization of DNMT1, promoter methylation-mediated repression of genes should be relieved in these cells. In other words, promoters of the genes upregulated in the KOs, should be GC-rich and should show a higher level of DNA methylation in WT cells. In order to verify this, we plotted GC density at differentially expressed genes against a random set of genes (Figure 3B). We observed that both up- and downregulated genes showed a much higher density of GC nucleotides compared with the control set of genes. In addition, we utilized previously published genome-wide DNA methylation levels in K562 cells to verify if the promoters of affected genes are methylated. As suspected, genes upregulated in KO cells show a much higher level of DNA methylation in K562 cells (Figure 3C), supporting the idea that gene expression changes we see in the KOs are due to changes in DNA methylation levels imposed by mis-localization of DNMT1 in the cytoplasm.

In order to further confirm that genes affected in the KO are regulated by DNMT1, we treated K562 cells with DNMT1 inhibitor, 5-aza-2′-deoxycytidine, also known as decitabine (DAC). DAC is a DNMT1 inhibitor that functions by covalently trapping DNMT1 to the DNA, thereby rendering it non-functional (Stresemann and Lyko, 2008). K562 WT cells were grown in the presence 0uM, 5uM, and 10uM DAC for 48 h. Treatment with both 5uM and 10uM DAC concentrations significantly reduced global 5mC levels (Figure 3D). DAC has also been shown to cause reductions in DNMT1 levels by inducing its proteasomal degradation (Ghoshal et al., 2005, 2018; Patel et al., 2010). Western blotting for DNMT1 also demonstrated decreased protein levels (Figure S4B).

Following confirmation of DNMT1 inhibition and global hypomethylation by DAC (Figure 3D), we next examined whether this elicited similar effects to that seen in *CCDC26* KO. Similar to the KOs, **γ**-H2AX immunofluorescence increased upon DNMT1 inhibition, indicating an increase in DNA damage in cells treated with both 5uM and 10uM DAC for 48 h (Figure S4C). Significantly, qRT-PCRs for DNMT1-regulated genes (Figure 3A) showed similar patterns of expression after DAC treatment as upon *CCDC26* KO (Figure 3E). Importantly, changes in gene expression were more pronounced in cells treated with 10uM DAC as compared with 5uM DAC (Figure 3E). These results support that reduction in DNMT1 can lead to DNA hypomethylation and subsequently DNA damage.

### Apoptosis and DNA damage are a consequence of DNMT1 mis-localization

We next sought to establish the sequence of events that results in DNMT1 mis-localizing to the cytosol and DNA hypomethylation in *CCDC26* KO cells. In order to fully understand the functional mechanism of *CCDC26*, it is important to examine whether cytosolic localization of DNMT1 is a consequence of DNA damage and apoptosis.

To further confirm that DNMT1 mis-localization is a result of *CCDC26* KO and not a consequence of DNA damage, it was critical to establish that this type of movement of DNMT1 is not a general response to DNA damage and apoptosis. To investigate this, DNA damage was induced in WT cells using cisplatin. Cisplatin is a platinum-based drug that forms bonds with, and ultimately crosslinks, bases within and between DNA strands. This can distort the double helix, interfere with both DNA replication and transcription, and consequently induce DNA damage and apoptosis (Goodsell, 2006). To begin, the amount of cisplatin and treatment time required to induce DNA damage but prior to complete cell death was optimized (Figure S5A). Microscopic observations demonstrated that after 24 h of cisplatin treatment, cells treated with 5uM or 10uM of the drug still appeared viable. DNA damage was also confirmed in the cells treated with 5uM and 10uM cisplatin by monitoring **γ**-H2AX foci using immunofluorescence (Figure S5B).

**Figure 4:**
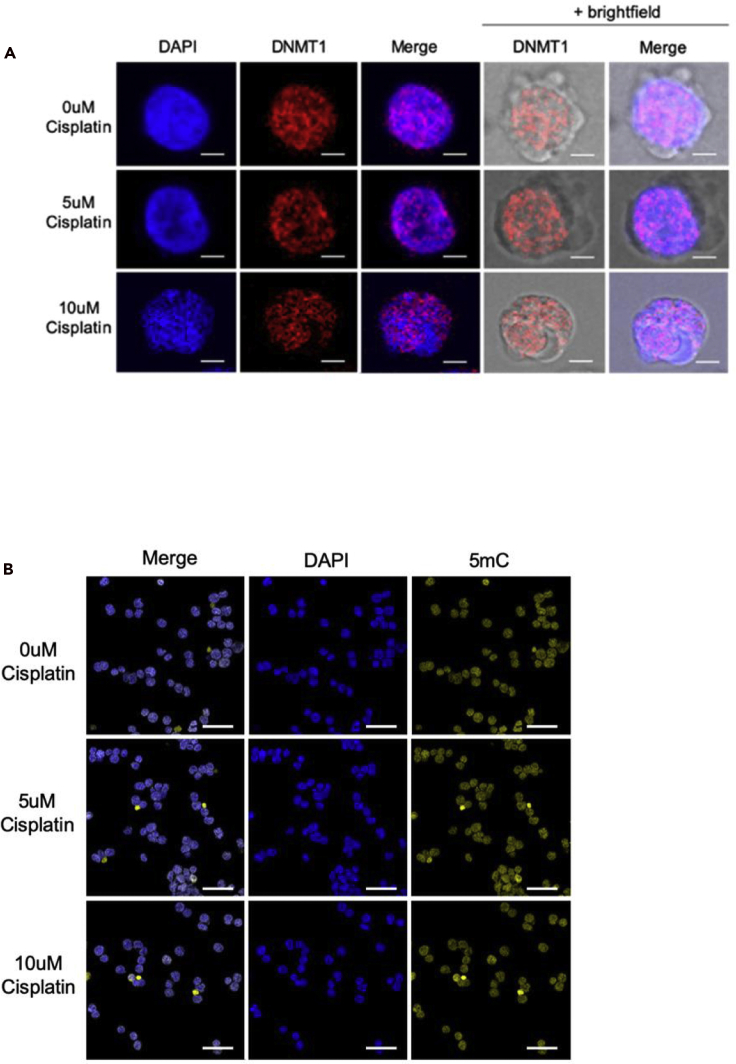
Cisplatin-induced DNA damage does not result in subcellular mis-localization of DNMT1 (A) Confocal images demonstrating the results of anti-DNMT1 immunofluorescence. Cells treated with 0 uM, 5 uM, and 10 uM cisplatin were stained with DAPI nuclear stain (blue) and anti-DNMT1 antibody (red). The outline of the cell membrane can be seen with the addition of the brightfield lens in the right-hand panels. DNMT1 appears nuclear in cells treated with different cisplatin concentrations. Scale bar, 5 um. (B) Confocal images demonstrating the results of anti-5mC immunofluorescence on cells treated with either 0 uM, 5 uM, or 10 uM cisplatin. Cells were stained with DAPI nuclear stain (blue) and anti-5mC antibody (yellow). Similar levels of 5mC fluorescence are observed in cells treated with different cisplatin concentrations. Scale bar, 50 um.

To assess DNMT1 localization in DNA-damage-induced cells, DNMT1 immunofluorescence was performed on cisplatin-treated cells. This experiment exhibited no significant differences between 0uM control cells and the drug-treated cells. Similar to WT K562 cells, in cisplatin-treated cells, DNMT1 appeared primarily nuclear, demonstrating a diffused pattern of distribution throughout (Figure 4A). Accordingly, there was also no substantial change in the levels of 5mC immunofluorescence between control and cisplatin-treated cells (Figure 4B). This suggests that DNMT1 mis-localization in cytoplasm is not a general consequence of DNA damage. As further confirmation, the expression levels of genes that are up- or downregulated in response to *CCDC26* KO and DNMT1 inhibition (as shown in Figure 3A) were also tested in cisplatin-treated cells. Some genes (*IGF1* and *CDKN1A*) demonstrated similar changes in expression patterns in response to cisplatin as they did in response to DNMT inhibition and *CCDC26* KO. However, for majority of genes, expression was either not significantly altered or they demonstrated an opposite change in expression (Figure S5C). This suggests that the expression changes seen in the KOs are response to DNMT1 mis-localization and DNA hypomethylation but not a consequence of DNA damage. Interestingly, *CDKN1A* gene whose promoter methylation was not affected by *CCDC26* KO (Figure S4A) was the only gene that was significantly upregulated in cisplatin-treated cells, indicating that changes in *CDKN1A* expression were in response to DNA damage downstream of DNA hypomethylation.

### *CCDC26* is a nuclear lincRNA and interacts with DNMT1

**Figure 5:**
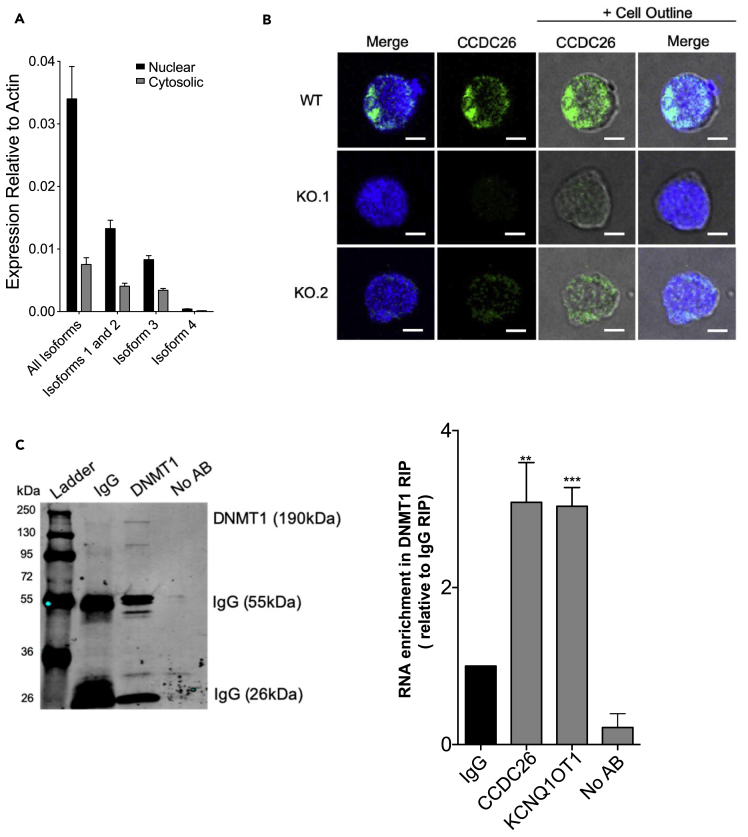
*CCDC26* is a nuclear lincRNA and interacts with DNMT1 (A) A plot showing the levels of different *CCDC26* isoforms, relative to Actin, in nuclear and cytosolic fractions of WT K562 cells, measured using qRT-PCR. Greater presence of all *CCDC26* isoforms was found in the nuclear fraction. Error bars represent the mean ± standard deviation. (B) Confocal images demonstrating the results of RNA FISH using a *CCDC26*-specific fluorescent probe. WT, KO.1, and KO.2 cells were stained with DAPI nuclear stain (blue) and a *CCDC26* probe (green). The outline of the cell membrane can be seen with the addition of the brightfield lens in the right-hand panels. *CCDC26* is primarily localized in the nucleus of the cell, specifically at the nuclear periphery. Scale bar, 5 um. (C) Protein-RNA complexes pulled down with either anti-IgG, anti-DNMT1, or no antibody were immunoblotted with anti-DNMT1, to ensure that the DNMT1 protein was correctly pulled down for RNA immunoprecipitation. RNA pulled-down in each IP was purified, converted to cDNA, and subjected to qRT-PCR with *CCDC26 and KCNQ1OT1* primers, to determine how much RNA was pulled down relative to the input in each instance. Values represent the mean ± standard deviation. ∗p < 0.05; ∗∗p < 0.01; ∗∗∗p < 0.001; NS = Not significant (unpaired, two-tailed t test).

To further investigate the relationship between *CCDC26* and DNMT1, we investigated the possibility that *CCDC26* interacts with DNMT1, thereby influencing its cellular localization. We first endeavored to find out the cellular localization of *CCDC26* RNA. A large proportion of lncRNAs that have shown a predominantly nuclear localization are either associated with chromatin or enriched in nuclear sub-compartments and organelles (Clemson et al., 2009; Hutchinson et al., 2007; Werner and Ruthenburg, 2015). The subcellular localization of a lncRNA can give a clue to its functional mechanism. Previous results showed that *CCDC26* might be marginally enriched in nucleus (Hirano et al., 2015). In this previous publication, all *CCDC26* isoforms were not tested for their localization. It is possible that only selected isoforms are nuclear, thus influencing *CCDC26* function. To understand the localization, we measured the levels of all four *CCDC26* isoforms using qRT-PCRs in nuclear and cytosolic RNA fractions (Figure 5A). snoRNA U105 and GAPDH were used as gene markers to assess the quality of nuclear and cytosolic fractions, respectively (Figure S6A). Actin was used as a housekeeping control gene against which *CCDC26* expression could be measured, given its similar levels in both the nucleus and cytosol (Figure S6B). Consistent with previous publication (Hirano et al., 2015), we found that all *CCDC26* isoforms were much more enriched in the nucleus as compared with the cytosol (Figure 5A). To determine the location of *CCDC26* in the nucleus, we also performed fluorescence *in situ* hybridization (RNA-FISH) on WT and KO cells. A fluorescently labeled probe specific to exon 6 of *CCDC26* was generated and used for RNA FISH, followed by analysis using confocal microscopy. Interestingly, microscopic images (Figure 5B) showed that *CCDC26* is predominantly located within the nucleus, demonstrating an enrichment at the periphery of the nucleus. The absence of fluorescent *CCDC26* signal in the KO cells indicated that the signal was specific (Figure 5B).

It is important to establish whether DNMT1 mis-localization could be due to a direct interaction between *CCDC26* and DNMT1 or an indirect effect of *CCDC26* knockout. DNMT1 has previously been shown to bind and undergo regulation by multiple lncRNAs (Bao et al., 2015; Chalei et al., 2014; Di Ruscio et al., 2013; Gao et al., 2020; Guo et al., 2019; Merry et al., 2015; Mohammad et al., 2010; O'Leary et al., 2017; Sun et al., 2016; Wang et al., 2015). It has also been suggested that DNMT1 has higher affinity for RNA than DNA (Di Ruscio et al., 2013; Merry et al., 2015). Nuclear localization of *CCDC26* points to a possibility that this lincRNA might also interact with DNMT1 in the nucleus. To further confirm this, we performed DNMT1 RNA immunoprecipitation (RIP) using anti-DNMT1 antibody (Figure 5C). An anti-IgG antibody was used to produce a control sample. The protein-bound-RNA pulled down with anti-DNMT1 and anti-IgG antibodies was used to perform qRT-PCRs with primers specific to *CCDC26*, and the RNA levels were measured relative to the input. Anti-DNMT1 pulled-down RNA showed approximately three times more enrichment of *CCDC26* compared with the IgG control (Figure 5C). Moreover, enrichment of *CCDC26* in the RIP assay is comparable to another long non-coding RNA, *KCNQ1OT1* (Figure 5C), which was previously shown to interact with DNMT1(Mohammad et al., 2010). This raises a possibility that *CCDC26* directly or indirectly interacts with DNMT1. However, the exact nature of *CCDC26* and DNMT1 interaction needs to be confirmed with a more detailed investigation.

However, we can get additional support for this interaction by analyzing previously studied protein-RNA interactions that have been studied using RIP or variations of this method (Bai et al., 2019; Di Ruscio et al., 2013; Hendrickson et al., 2016; Hui et al., 2019; Xu et al., 2019). At least two datasets exploring DNMT1-RNA interactions in myeloid cells have been published (Di Ruscio et al., 2013; Hendrickson et al., 2016). We first adopted a bioinformatics approach where we re-mapped previously published RIP-seq datasets to the *CCDC26* locus. The first dataset that we analyzed was generated using another myeloid line, HL60 (Di Ruscio et al., 2013). These data were generated by first pulling down cellular RNAs that bind to DNMT1 using anti-DNMT1 antibody and then sequencing and mapping these RNAs to the human genome. These data showed that *CCDC26* is highly enriched in DNMT1-RIP assays when compared with an IgG RIP control (Figure S6C). Furthermore, analysis of another dataset, produced by a variation of the RIP method, formaldehyde-RIP-seq (fRIP-seq), also showed high enrichment of *CCDC26* in DNMT1-bound RNAs in K562 cells (Hendrickson et al., 2016), further confirming an interaction between DNMT1-*CCDC26* (Figure S6D).

## Discussion

In the last three decades, following the development of whole-genome technologies, lncRNAs have gained importance (Derrien et al., 2012; Hangauer et al., 2013; Iyer et al., 2015; Okazaki et al., 2002), with many examples demonstrating their role in transcription regulation.

Here we have performed functional analysis of lincRNA, *CCDC26* and demonstrated its importance in regulating global DNA methylation. Our data show that, in the absence of *CCDC26*, the genome is hypomethylated, which leads to increase in apoptosis and DNA damage and cell growth inhibition. In the past, non-coding RNAs have been shown to impact DNA methylation levels through transcriptional and post-transcriptional regulation of DNMT genes (Chen et al., 2015; Cheng et al., 2018; Di Ruscio et al., 2013; Merry et al., 2015; Mohammad et al., 2010; Wang et al., 2018). However, DNMT expression levels were unchanged in *CCDC26* KOs, indicating that the mechanism behind the observed DNA hypomethylation is different. Strikingly, in the absence of lincRNA *CCDC26*, a large proportion of DNMT1 protein is mis-localized in the cytosol. In KO cells, the mis-localization of DNMT1 in cytoplasm is most likely responsible for the observed hypomethylated state of the genome and cell death. This connection between *CCDC26* and DNMT1 can provide the missing link between frequent mutations in *CCDC2*6 locus and leukemia. Deletion of DNMT1 is shown to prevent MLL-AF9 leukemia. In addition, it has been reported that the absence of DNMT1 induces apoptosis in hematopoietic stem cells. This is similar to what we observe in this study when *CCDC26* is removed. In corollary, direct or indirect role of *CCDC26* in retaining DNMT1 in nucleus and also maintaining cell proliferation can explain why *CCDC26* is often upregulated in AML (Duployez et al., 2018; Izadifard et al., 2018; Kuhn et al., 2012; Radtke et al., 2009).

LncRNA-mediated regulation of cellular localization, although not reported in case of DNMT1, has been previously reported in case of other proteins. In some instances, this has been shown to occur via a direct interaction; for example, lncRNA *TP53TG1* binds the transcription factor, YBX1, thereby preventing its nuclear trafficking (Diaz-Lagares et al., 2016). In other instances, this can also occur via an indirect effect; an interaction between lncRNA *CRYBG3* and actin for example is sufficient to prevent translocation of myelin and lymphocyte protein (MAL) into the nucleus (Pei et al., 2018). Similarly, NF_k_B-interacting LncRNA (*NKILA*) binds and prevents phosphorylation of the inhibitory IκB subunit. This blocks its degradation, which subsequently prevents the active p65 subunit of NF_k_B from re-localizing from the cytosol to the nucleus (Liu et al., 2015). However, we did not observe any changes in DNMT1 stability upon *CCDC26* KO (Figure S7). Moreover, we demonstrate an interaction between DNMT1 and *CCDC26*, suggesting that this lincRNA might play a direct or indirect role in localizing DNMT1 in the nucleus.

**Figure 6:**
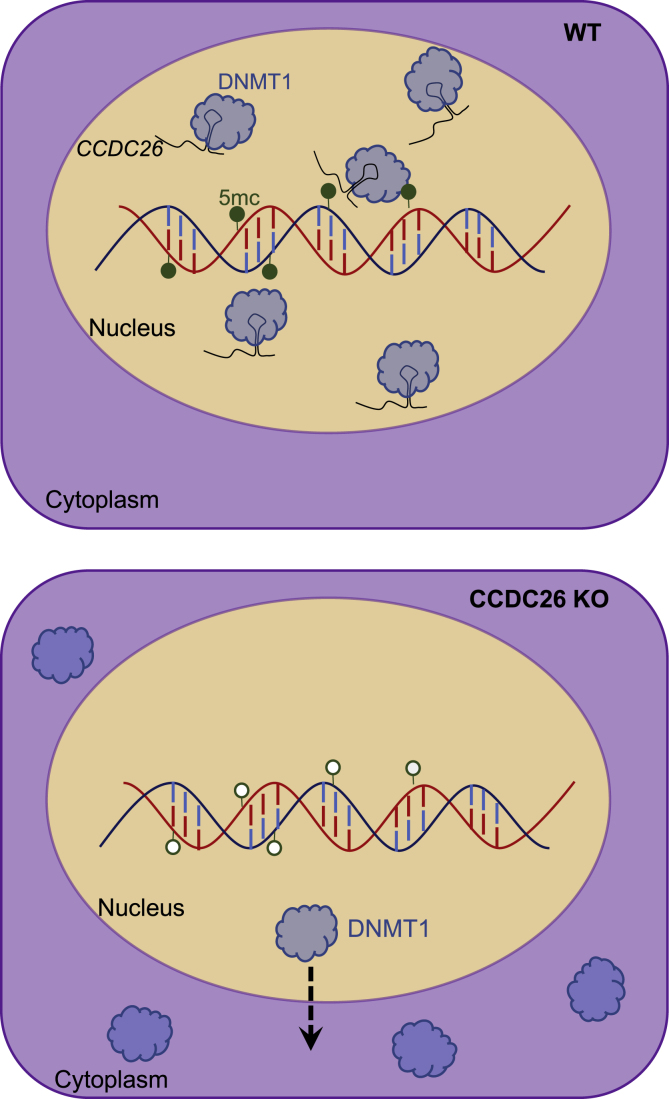
Model for *CCDC26*-mediated DNMT1 regulation In WT K562 cells, DNMT1 directly or indirectly interacts with *CCDC26*. DNMT1 is almost exclusively localized in the nucleus where it maintains DNA methylation patterns as cells replicate. In the absence of *CCDC26*, DNMT1 is re-localized to the cytoplasm and cells become hypomethylated.

Numerous DNMT1-interacting lncRNAs have been identified previously (Bao et al., 2015; Chalei et al., 2014; Di Ruscio et al., 2013; Gao et al., 2020; Guo et al., 2019; Merry et al., 2015; Mohammad et al., 2010; O'Leary et al., 2017; Sun et al., 2016; Wang et al., 2015); however, these largely demonstrate effects on DNA methylation at localized genes or regions, as opposed to the global effect observed here. In addition, lincRNA-mediated DNMT1 localization has not been reported before. Based on these results, we provide a model (Figure 6) suggesting that lincRNAs, in this case *CCDC26*, can regulate sub-cellular localization of DNMT1 via direct or indirect interaction in the nucleus. This provides a means of regulating global genomic DNA methylation, and disruption of the lincRNA can result in hypomethylation and apoptosis.

A major question that remains in this instance is how *CCDC26* orchestrates nuclear localization of DNMT1 and the mechanism that causes its cytosolic mis-localization in the absence of *CCDC26*. Independent of lincRNAs, in rare circumstances, DNMT1 localization in the cytoplasm has been demonstrated and can provide clues regarding the mechanism behind lincRNA-mediated DNMT1 localization. Arguably the best studied of these instances is during preimplantation in early development. An oocyte-specific form of DNMT1, DNMT1o, demonstrates a preferential localization within the cytosol during preimplantation development. DNMT1o lacks 118 amino acids at the N-terminus compared with somatic DNMT1. It has been postulated that an alternative, extended region of the N-terminus is critical, and complex folding of this area likely plays a large part in overriding the NLS. This is required for demethylation of the embryonic genome, upon which lineage specific methylation patterns are established (Cardoso and Leonhardt, 1999). In instances other than during embryonic development, cytosolic localization of DNMT1 tends to be aberrant; for example, it has been associated with several neurological disorders including hereditary sensory and autonomic neuropathy type 1E (HSAN1E) (Baets et al., 2015), Alzheimer disease (Mastroeni et al., 2013) and Parkinson disease (Desplats et al., 2011), as well as cancer tumorigenesis (Arzenani et al., 2011; Hodge et al., 2007). The reasons behind aberrant cytosolic localization of DNMT1 is not entirely clear. Various mechanisms including changes to post-translational modifications of DNMT1 (Hodge et al., 2007), HDAC inhibition (Arzenani et al., 2011), mutations within the RFTS domain (Baets et al., 2015), and disruption to nucleo-cytoplasmic transport systems across the nuclear membrane (Mastroeni et al., 2013) have been reported. Given that previous reports of cytosolic DNMT1 have often involved the N-terminal domain of DNMT1 (Baets et al., 2015; Cardoso and Leonhardt, 1999; Hodge et al., 2007), where the NLS resides, it can be speculated that this region of the protein may be affected by *CCDC26*, possibly through lincRNA-mediated post-translational modifications or protein folding.

### Limitations of the study

Although our study shows that *CCDC26* influences DNMT1 localization, to gain further insights into how *CCDC26* functions, we will have to address a number of questions and will need to carry out a more detailed investigation. Our RIP assay indicates that *CCDC26* is similarly enriched in DNMT1 pulldown as other ncRNAs that are previously reported to bind to DNMT1. However, we will need to confirm if this is a direct or indirect interaction. We can speculate that the interaction of *CCDC26* with DNMT1 leads to changes in the configuration of DNMT1's nuclear localization domain that are needed for retaining DNMT1 in the nucleus. RNA-binding domain of DNMT1 is previously mapped, and it overlaps its C-terminal enzymatic domain (Di Ruscio et al., 2013). We will need to verify that *CCDC26* binds to the same domain, and also, we will need to investigate the relationship between *CCDC26* binding domain and nuclear localization domain of DNMT1. If *CCDC26* and DNMT1 interact *in vivo* we expect them to colocalize in the cell. Our microscopy observations show that both DNMT1 and *CCDC26* are nuclear. But, in addition, we have also observed that *CCDC26* also accumulates in the nuclear periphery. We have not studied the significance of accumulation of *CCDC26* in the nuclear periphery. However, our observation that *CCDC26* knockout does not affect localization of other nuclear proteins such as EZH2 and HDAC2 shows that the role of *CCDC26* is specific and it does not affect all nuclear proteins. Lastly, it is important to see if DNMT1 mis-localization is reversible and if re-introducing *CCDC26* in the cells will re-localize DNMT1 in the nucleus. Currently, many of these questions are out of scope of this study but will need to be addressed in the future.

In summary, even though detailed molecular mechanism behind *CCDC26*-mediated DNMT1 localization still remains to be investigated, our study provides an insight into the role of *CCDC26* in cancer as well as a novel lincRNA mechanism of DNMT1 regulation.

### Resource availability

#### Lead contact

Further information and requests for resources and reagents should be directed to and will be fulfilled by the lead contact, Aditi Kanhere (a.kanhere@liverpool.ac.uk).

#### Materials availability

This study did not generate new unique reagents.

#### Data and code availability

The accession number for the RNA-sequencing data generated and reported in this paper is GEO: GSE105029.

## Methods

All methods can be found in the accompanying transparent methods supplemental file.

## Supplemental information

Document S1. Transparent methods, Figures S1–S7, and Tables S1–S5
