# Supplementary material for: A long intergenic non-coding RNA regulates nuclear localization of DNA methyl transferase-1
Source: iScience. 2021 Mar 5;24(4):102273. doi: 10.1016/j.isci.2021.102273 (PMC8022221; doi:10.1016/j.isci.2021.102273)
Supplement: Document S1. Transparent methods, Figures S1–S7, and Tables S1–S5 [file mmc1.pdf]

iScience, Volume *24*

Supplemental information

A long intergenic non-coding RNA regulates nuclear localization of DNA methyl transferase-1

Rhian Jones, Susanne Wijesinghe, Claire Wilson, John Halsall, Triantafillos Liloglou, and Aditi Kanhere

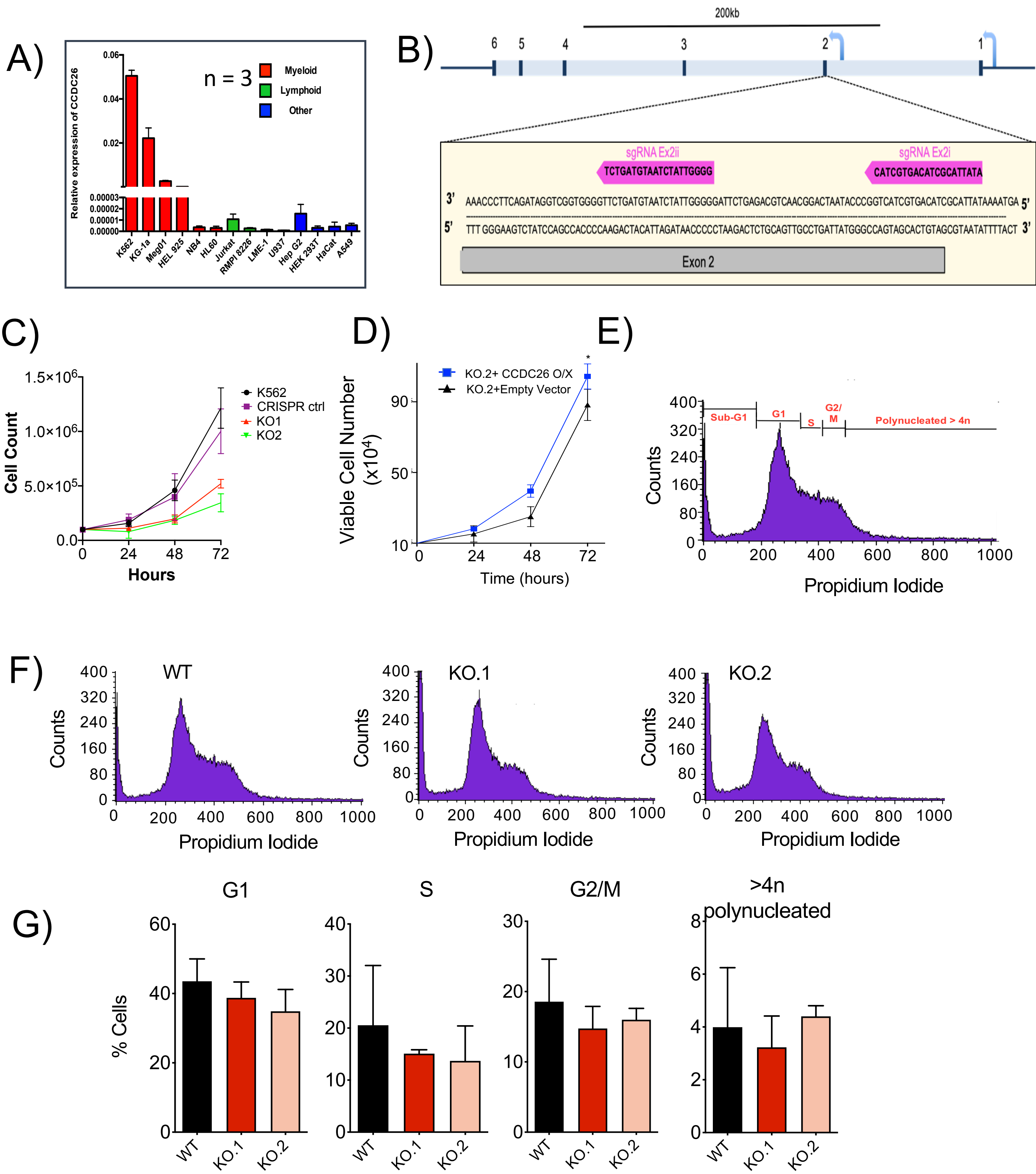

Figure S1.The effect of CCDC26 knockout on cell growth and cell cycle, Related to Figure 1

(A) *CCDC26* expression in 14 different cell-lines of diverse origins. Expression was measured using qRT-PCR assays and normalised against expression levels of Actin.

(B) Schematic of the *CCDC26* gene showing that TSS2 was targeted with two sgRNAs simultaneously using CRISPR/Cas9 technology to establish *CCDC26* KO cell lines.

(C) Growth curves for K562, CRISPR control and CCDC26 knockouts.

(D) Growth curves of KO.2 expressing CCDC26 from an exogenous vector (CCDC26 o/x) and empty vector.

(E) A schematic showing how the number of cells in different cell cycle stages are calculated after FACS analysis.

(F) Histograms showing distribution of WT and KO cells in different cell cycle stages after propidium iodide staining and FACS analysis.

(G) Plots showing the percentage of WT, KO.1 and KO.2 cells in each stage of the cell cycle following propidium iodide staining and FACS analysis. Values represent the mean ± standard deviation (n=3) (unpaired, two-tailed *t* test).

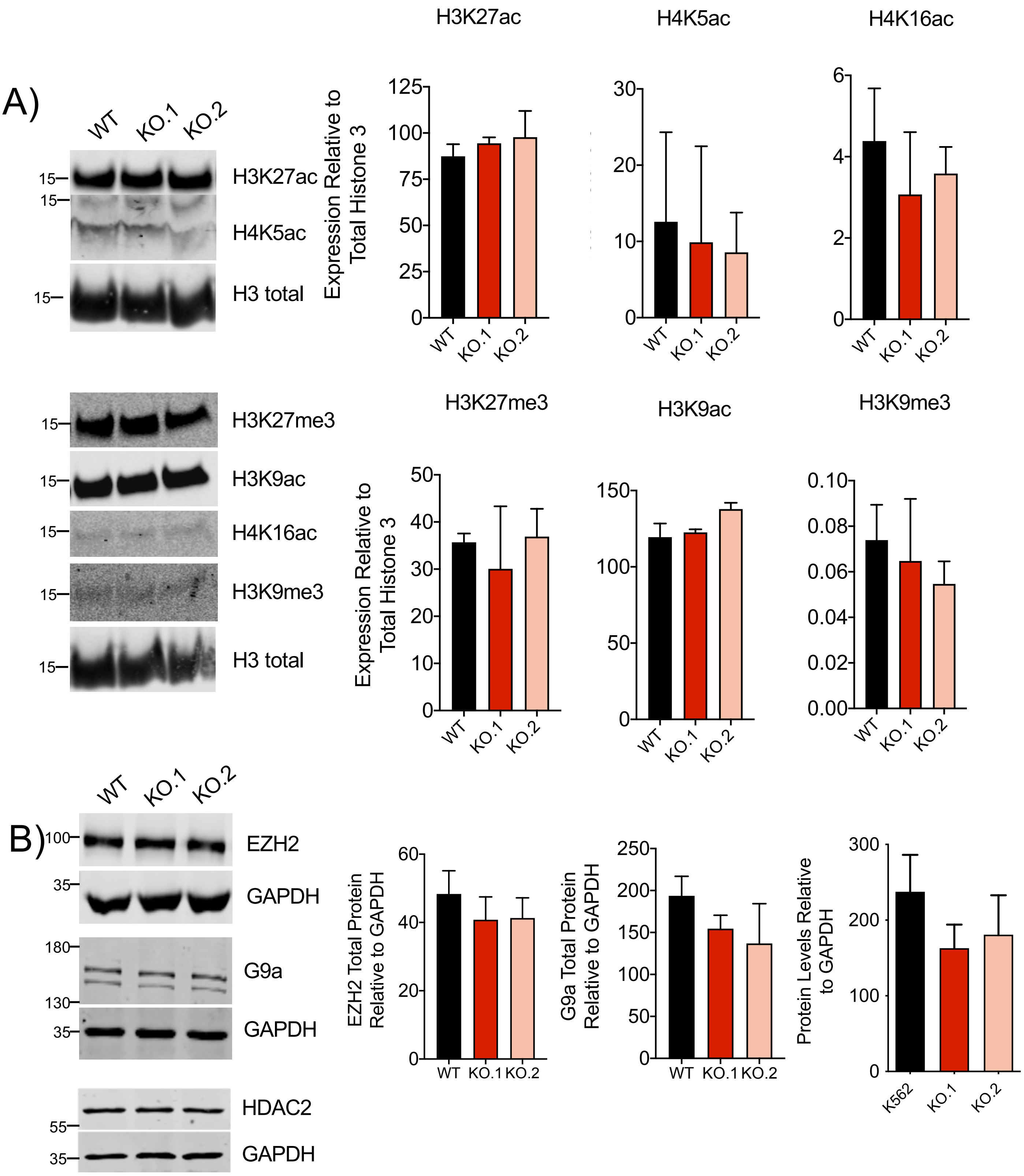

Figure S2. CCDC26 knockout has no effect on genomic level of histone modifications, Related to Figure 2

(A) Immunoblotting on histone protein isolated from WT and CCDC26 KO cells using antibodies against common histone modifications (anti-H3K27ac, -H4K5ac, -H3K27me3, -H3K9ac, -H4K16ac and H3K9me3). Levels were measured relative to total histone H3 levels. Values represent the mean ± standard deviation (n=3) (unpaired, two-tailed *t* test).

(B) Total protein levels of histone modifying enzymes, EZH2, G9a and HDAC2 measured relative to GAPDH by immunoblotting, are unchanged in WT and *CCDC26* KO cells. Values represent the mean ± standard deviation (n=3) (unpaired, two-tailed *t* test).

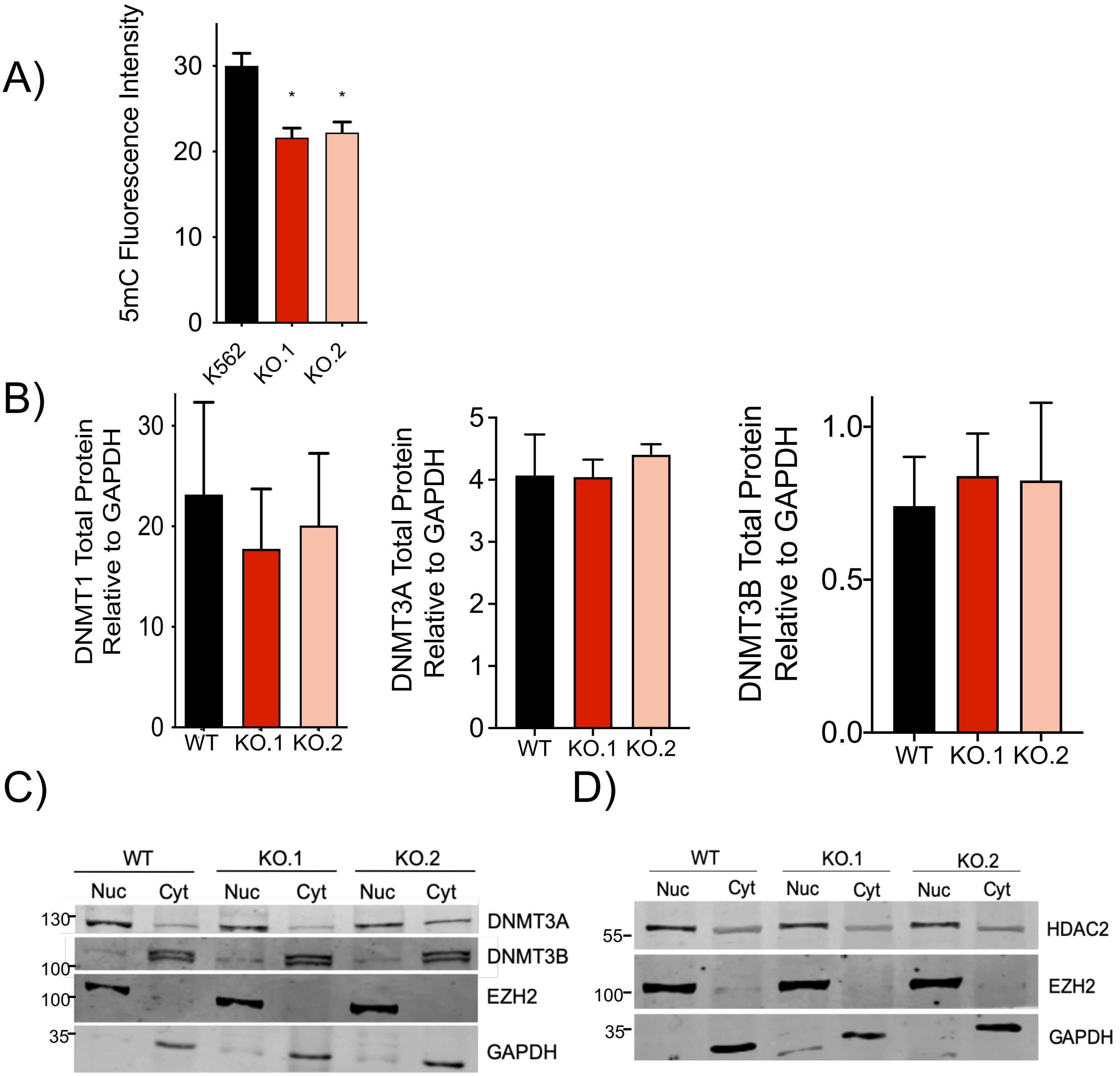

Figure S3. CCDC26 removal leads to DNA hypomethylation but has no effect on DNMT levels, Related to Figure 2

(A) 5mC Immunofluorescence intensity measurements in *CCDC26* KO cells compared to WT. 5mC fluorescence intensity was measured in 2D confocal images for 200 individual nuclei, per replicate, using FIJI image analysis software. Values represent the mean ± standard deviation (n=3). * P<0.05 (unpaired, two-tailed *t* test).

(B) Plots showing total protein levels of DNMT1, DNMT3a and DNMT3b relative to GAPDH in WT K562, KO.1 and KO.2 cells by immunoblotting. Values represent the mean ± standard deviation. * P<0.05 (unpaired, two- tailed *t* test) (n=3).

(C) Immunoblotting for DNMT3A and DNMT3B on nuclear and cytosolic protein fractions show no significant difference in subcellular localization between WT and *CCDC26* KO cells. EZH2 and GAPDH are used as nuclear and cytosolic markers respectively (nuc = nuclear protein fraction; cyt = cytosolic protein fraction).

(D) Immunoblotting for HDAC2 on nuclear and cytosolic protein fractions show no significant difference in subcellular localization between WT and *CCDC26* KO cells. EZH2 and GAPDH are used as nuclear and cytosolic markers respectively (nuc = nuclear protein fraction; cyt = cytosolic protein fraction).

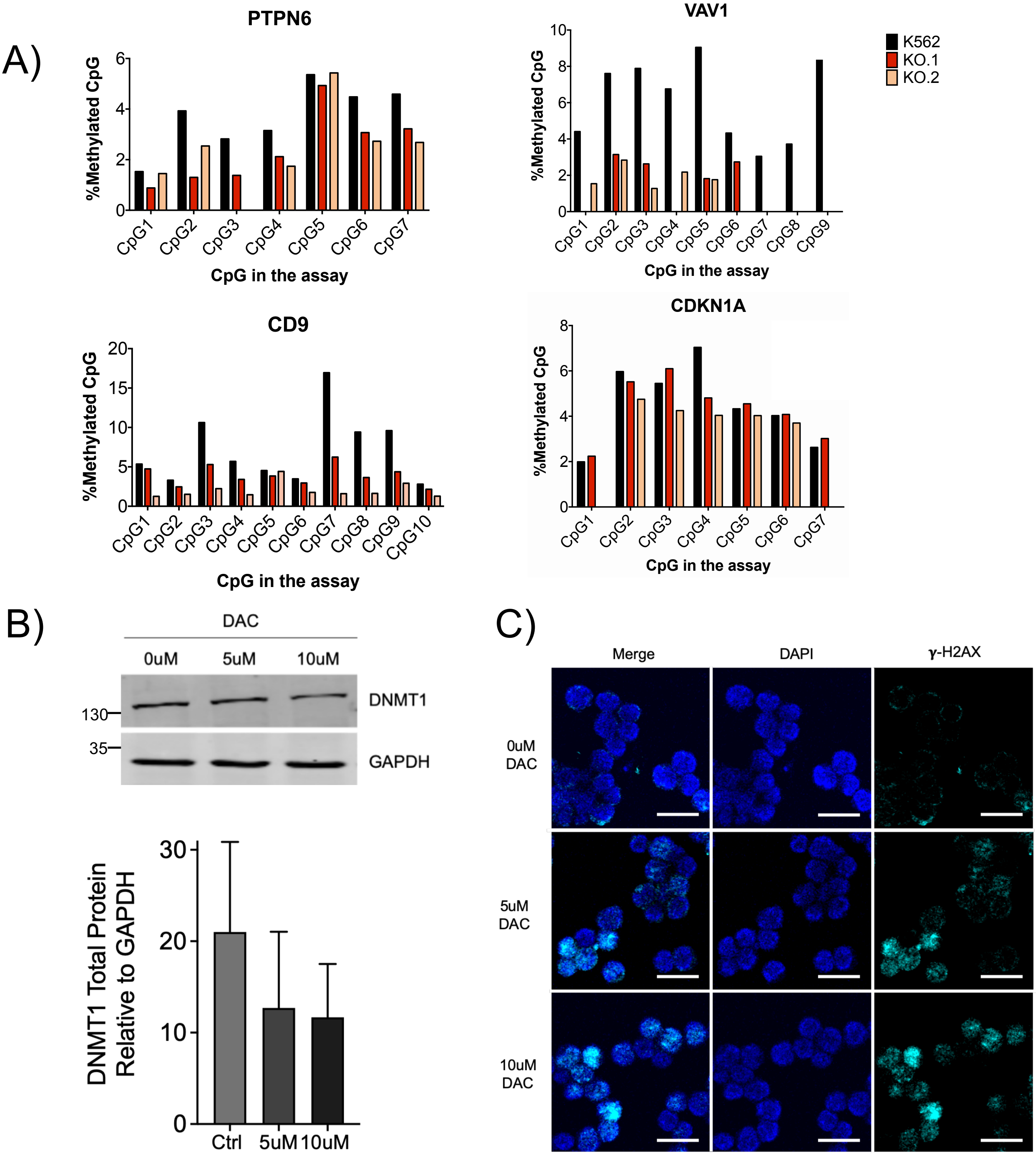

Figure S4. DNMT1 inhibition by DAC has the same effect as CCDC26 knockout, Related to Figure 3

(A) DNA methylation profiling at individual gene promoters using bisulphite conversion and pyrosequencing. Percentage of methylated CpGs in individual promoters in K562 and the two knockouts is plotted as a bar chart.

(B) Immunoblotting for DNMT1 total protein levels in cells treated with 0uM 5uM and 10uM DAC, measured relative to GAPDH. DNMT1 levels are slightly reduced in cells treated with 5uM and 10uM DAC. Values represent the mean ± standard deviation (n=3) (unpaired, two-tailed *t* test).

(C) Confocal images demonstrating the results of anti-**γ**-H2AX immunofluorescence. Cells treated with 0uM, 5uM and 10uM DNMT1 inhibitor, DAC, were stained with DAPI nuclear stain (blue) and anti-**γ**-H2AX antibody (cyan). Increased numbers of **γ**-H2AX foci are present in the cells treated with 5uM and 10uM DAC Scale bar = 25um.

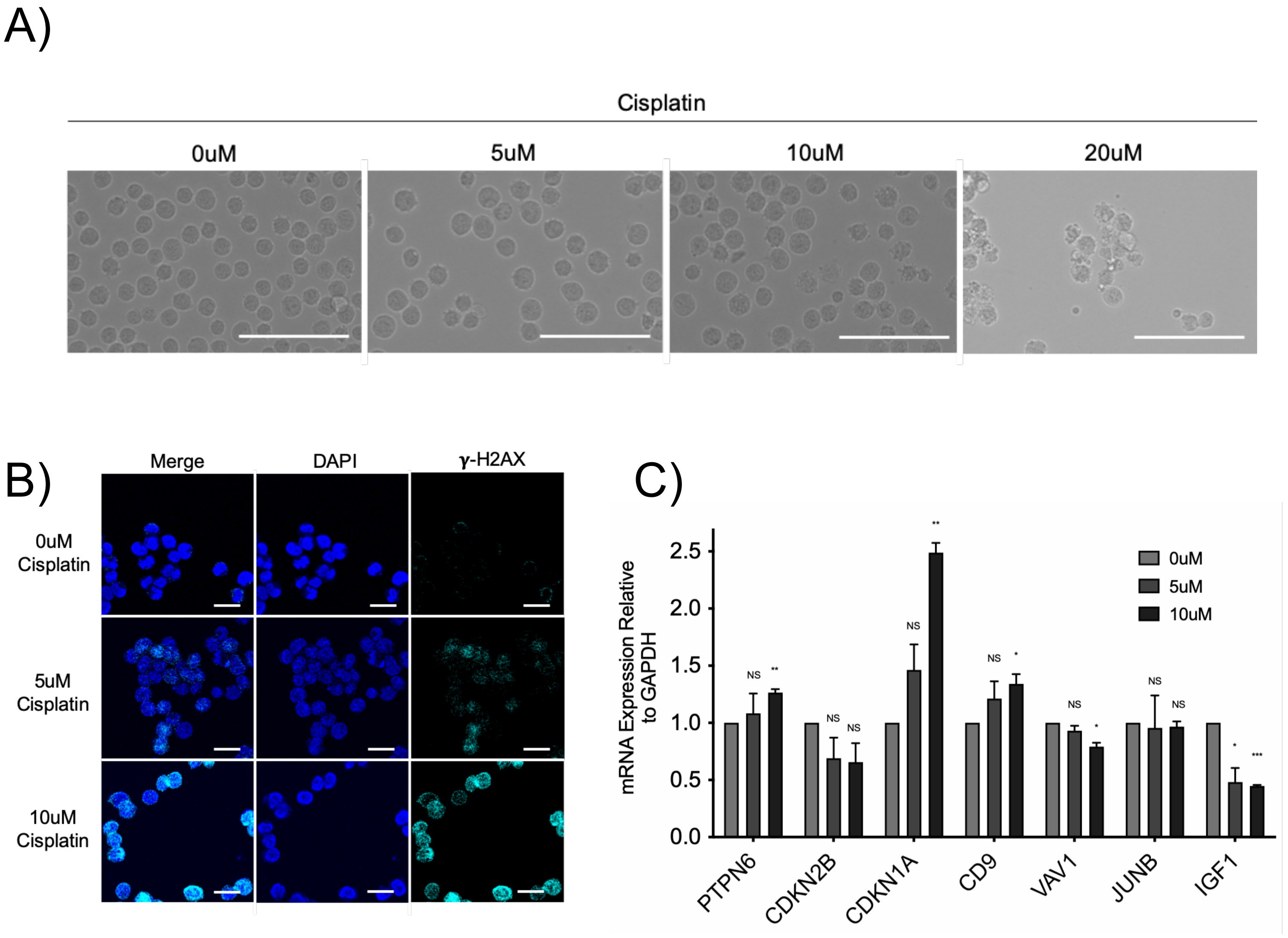

Figure S5. Cisplatin induced DNA damage has no effect on DNMT1 regulated genes, Related to Figure 4

A) Brightfield microscopy images of K562 cells treated with increasing concentrations of DNA damage-inducing drug, cisplatin. Cells appear increasingly distressed with increasing cisplatin concentrations. Scale bar = 100um.

B) Confocal images demonstrating the results of anti-**γ**-H2AX immunofluorescence. Cells treated with 0uM, 5uM and 10uM DNA damage-inducing drug, cisplatin, were stained with DAPI nuclear stain (blue) and anti-**γ**-H2AX antibody (cyan). Increased numbers of **γ**-H2AX foci are present in the cells treated with 5uM and 10uM cisplatin. Scale bar = 25um.

C)A plot showing expression levels of various genes whose expression have previously been shown to be impacted by DNMT1 depletion or DNA hypomethylation in myeloid leukemia. Levels are measured relative to GAPDH in cells treated with 0uM, 5uM and 10uM cisplatin. Values represent the mean ± standard deviation. * P<0.05; **P<0.01; ***P<0.001; NS = Not significant (unpaired, two- tailed *t* test).

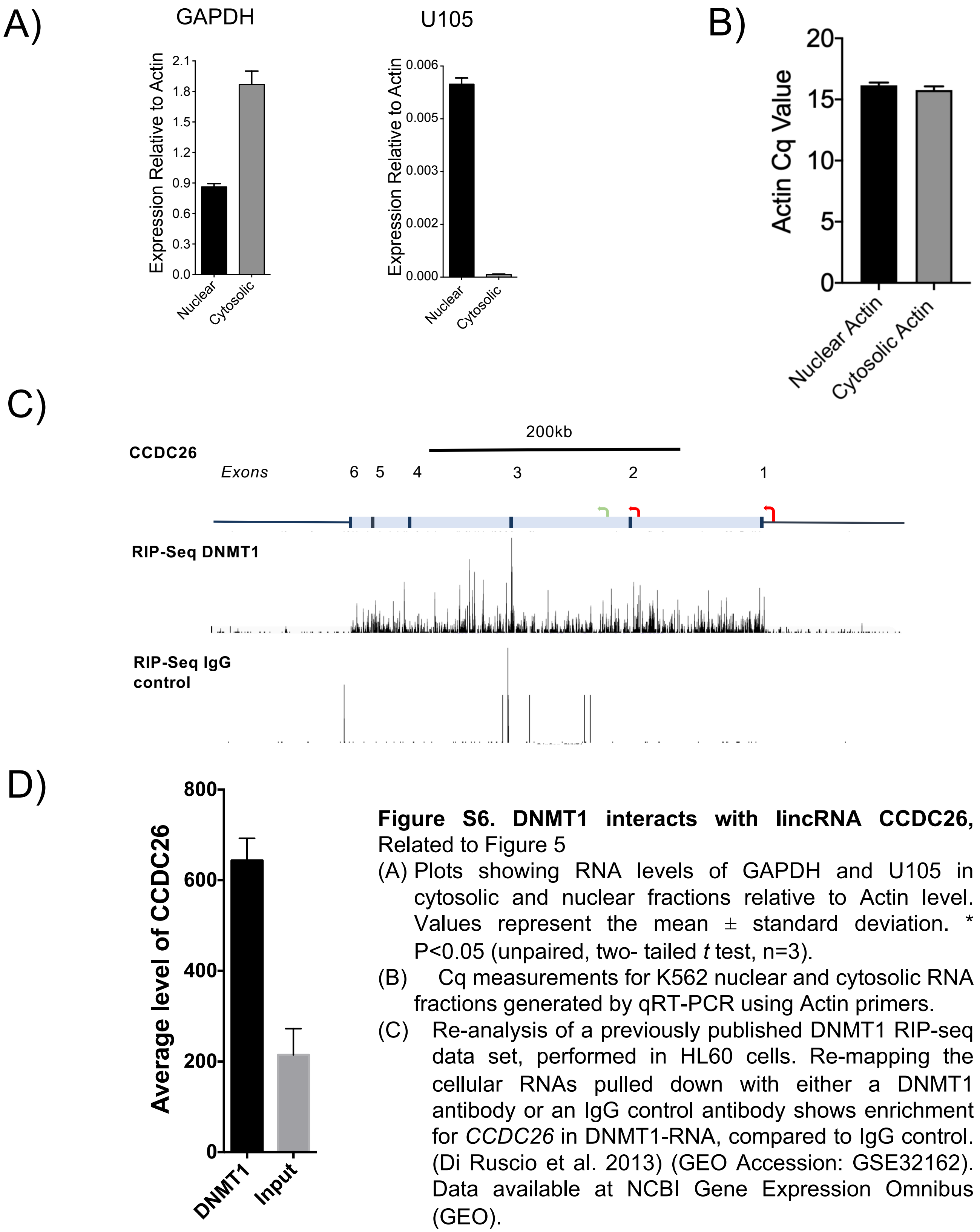

Figure S6. DNMT1 interacts with lincRNA CCDC26, Related to Figure 5

(A) Plots showing RNA levels of GAPDH and U105 in cytosolic and nuclear fractions relative to Actin level. Values represent the mean ± standard deviation. * P<0.05 (unpaired, two- tailed *t* test, n=3).

(B) Cq measurements for K562 nuclear and cytosolic RNA fractions generated by qRT-PCR using Actin primers.

(C) Re-analysis of a previously published DNMT1 RIP-seq data set, performed in HL60 cells. Re-mapping the cellular RNAs pulled down with either a DNMT1 antibody or an IgG control antibody shows enrichment for *CCDC26* in DNMT1-RNA, compared to IgG control. (Di Ruscio et al. 2013) (GEO Accession: GSE32162). Data available at NCBI Gene Expression Omnibus (GEO).

(D) Re-analysis of a previously published DNMT1 fRIP-seq data set, performed in K562 cells shows high enrichment of *CCDC26* in DNMT1-bound RNAs (Hendrickson et al. 2016) (GEO Accession: GSE67963). Data available at NCBI Gene Expression Omnibus (GEO).

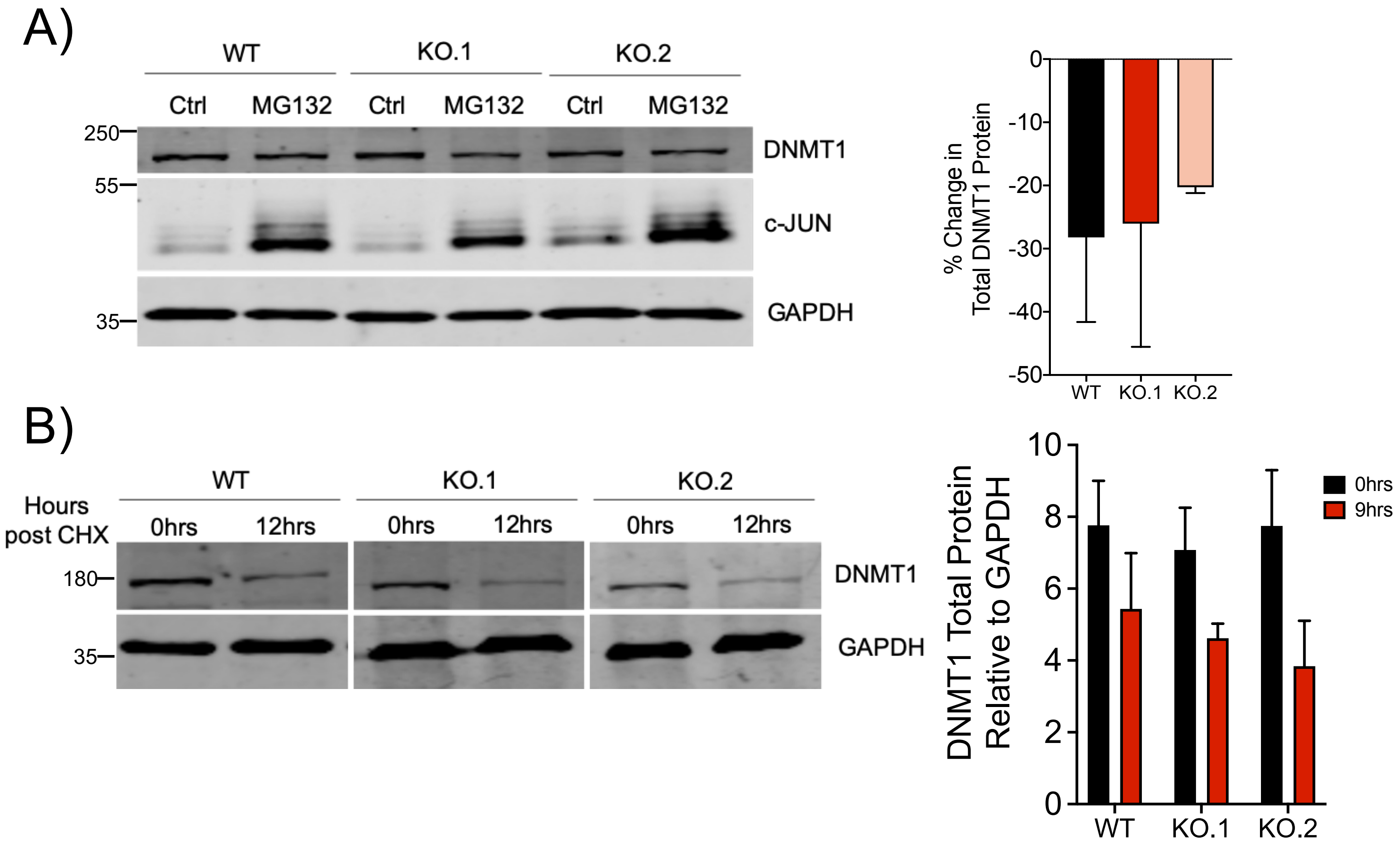

Figure S7. DNMT1 stability is not affected by CCDC26 knockout, Related to discussion in main text.

(A) Immunoblotting for DNMT1 total protein levels in WT and CCDC26 KO cells following treatment with 10uM of proteosomal inhibitor MG132. DNMT1 protein levels fell in both WT and KO cells. The difference in the extent to which DNMT1 levels fall was not statistically significant between cell lines. Immunoblotting for c-JUN was also performed as a control to show that the MG132 inhibitor was working. C-JUN levels rose upon MG132 treatment in both WT and KO cells. Protein levels were measured relative to the housekeeping protein, GAPDH. Values represent the mean ± standard deviation (unpaired, two-tailed *t* test, n=3).

(B) Immunoblotting for DNMT1 total protein levels on WT and CCDC26 KO cells treated with cycloheximide (CHX) for 0hrs and 12hrs. The difference in the extent to which DNMT1 levels fall was not statistically significant between WT and KO cells. Values represent the mean ± standard deviation (unpaired, two-tailed *t* test, n=3).

Table S1. Primers, related to figure 1, figure 3 and figure 5

All primers were purchased from Sigma and are shown 5'-3'

Primer	Forward	Reverse
CCDC26_RNAFISH	CCTGCCACACTGGGAAAGAT	GAAATTAATACGACTCACTATAGTTGACTTCCCA GGAGCGAA
CCDC26_All (all isoforms)	ATGGAAAGATTGTGCCTGCAG	CTCGATCTTTCCCAGTGTGG
CCDC26_Set1-2 (isoforms 1 and 2)	TCAGGCAACTGCAGAGTCTTAG	ACCCAGGCTTGTCTCATCTC
CCDC26_Set3 (isoform 3)	AGATCAGCTATGAAGGCCTGAG	CTCGATCTTTCCCAGTGTGG
CCDC26_Set4 (isoform 4)	TTCAAGAATGGCCTTTTAAAGGACC	CTCGATCTTTCCCAGTGTGG
GAPDH	GGAGCGAGATCCCTCCAAAAT	GGCTGTTGTCATACTTCTCATGG
U105	CCCTATCTCTCATGATGAAC	CCCATCTCTTCTTCAGAGCG
DNMT1	GCGGTATACCCACCATGACA	AGGCTTTGCCGGCTTCC
DNMT3A	GTTGTGAGAAGGAATGGGCG	TTGGCTTTCTTCTCAGCCGTAT
DNMT3B	CACTCTGTCCTGGGTGCTG	GTCTCCCTTCATGCTTTCCAAG
PTPN6	AACAGCCGTGTCATCGTCAT	ATCAGGTCTCCATTGTCCAGC
CDKN2B	TGGGGGCGGCAGCGATGAG	AGGTGGGTGGGGGTGGGAAAT
CDKN1A	AGCATGACAGATTTCTACCACTC	GATGTAGAGCGGGCCTTTGA
CD9	CATCTGTATCCAGCGCCAGG	CCGGCAAGCCAGAAGATGAA
VAV1	CTTACGGAGCTGGTGGAGTT	ACTTTGTGCTTCCCACTGCT
JUNB	CTGCTGGAAACAGACTCGATTC	CCACAGTACGGTGCAGAGAG
IGF1	TGCTCTCAACATCTCCCATCTC	TGGTGTGCATCTTCACCTTCA
Actin B	TACTCCTGCTTGCTGATCCA	GATCATTGCTCCTCCTGAGC

Table S2. Primary Antibodies, related to figure 1, 2, 3, 4 and 5

WB = western blot; IF = immunofluorescence; IP = immunoprecipitation; RIP = RNA immunoprecipitation; M = monoclonal; P = polyclonal

Antibody	Use	Working Dilution	Origin	Company	Catalog No.	Clonality
GAPDH	WB	1:1000	Mouse	ThermoFisher Scientific	MA5-15738	M
DNMT1	WB	1:1000	Mouse	NovusBio	NB100-56519	M
DNMT3A	WB	1:1000	Rabbit	NovusBio	NB100-265SS	P
DNMT3B	WB	1:1000	Rabbit	BioVision	3275	P
EZH2	WB	1:1000	Rabbit	Cell Signaling	#5246	M
HDAC2	WB	1:700	Rabbit	Santa Cruz	sc-7899	P
Caspase 9	WB	1:1000	Rabbit	Cell Signaling	#9508	M
H3K27ac	WB	1:1000	Rabbit	Abcam	ab4729	P
H4K5ac	WB	Generated and kindly gifted by Dr John Halsall (Turner Group)				
H3K27me3	WB	1:1000	Mouse	Abcam	ab6002	M
H3K9ac	WB	Generated and kindly gifted by Dr John Halsall (Turner Group)				
H4K16ac	WB	1:1000	Rabbit	Abcam	ab109463	M
H3K9me3	WB	1:500	Rabbit	Millipore	07-442	P
Histone H3	WB	1:5000	Rabbit	Abcam	ab1791	P
c-JUN	WB	1:1000	Rabbit	Cell Signaling	#9165	M
DNMT1	IF	1:10	Mouse	NovusBio	NB100-56519	M
5mC	IF	1:250	Mouse	Epigentek	A-1014	M
γ-H2AX	IF	1:100	Mouse	Millipore	05-636-AF555	M
DNMT1	RIP	/	Mouse	NovusBio	NB100-56519	M
IgG	RIP	/	Mouse	Sigma	18765-5MG	/

Table S3. Secondary Antibodies, figure 1, 2, 3, 4 and 5
WB = Western Blot; IF = Immunofluorescence

Antibody	Use	Working Dilution	Company	Catalog No.
**Anti-rabbit IgG (H+L) (DyLight™ 800 4X PEG Conjugate)**	WB	1:10,000	Cell Signaling	5151
**Anti-mouse IgG (H+L) (DyLight™ 680 Conjugate)**	WB	1:10,000	Cell Signalling	5470
**Alexa Fluor 488 donkey anti-rabbit IgG (H+L)**	IF	1:500	Invitrogen	A21206
**Alexa Fluor 633 goat anti-mouse IgG (H+L)**	IF	1:500	Invitrogen	1010093

Table S4: Sequences of primers for Pyrosequencing Methylation Analysis, related to figure 3

Primer name	Sequence 5'-> 3'	Modification
VAV1meth-F	GGTAAAGAAGAGGAAGTGGTA	
VAV1meth-Rb	AACTCCACAACTCCATAACTAC	5' biotin
VAV1meth-S	AGAAGAGGAAGTGGTAGTATT	
IGF2meth-Fb	GAAGGATATAATTTTGTTGAGAA	5-biotin
IGF2meth-R	TCCCTTTAAATAAATCTAACTACTA	
IGF2meth-S	AACTACTACTATCAATACACC	
CD9meth-F	GGGGAAGAGTTTTTTAAAGTAG	
CD9meth-Rb	CACTCCCTACCACTTTTACC	5' biotin
CD9meth-S	AAGAGTTTTTTAAAGTAGAA	
CDKN1Ameth-Fb	TTAGTGGGGAAATGTGTTTA	5' biotin
CDKN1Ameth-R	CAAACCCAAACTCCTAACTAC	
CDKN1Ameth-S	CCCAAACTCCTAACTACC	
PTPN6meth1-F	AGTTTTTAGGAAAAGGATAGG	
PTPN6meth1-Rb	TCCAAATAACTCCTCCTCTC	5' biotin*
PTPN6meth1-S	TTTAGGAAAAGGATAGGTG	

F: forward PCR primer, R: Reverse PCR primer, S: pyrosequencing primer, b: biotinylated

Table S5. Pyrosequencing Methylation Analysis, related to figure 3

Promoter	Cell line	Mean Rank	p-value
CD9	K562	3	<0.001
	KO.1	1.9	
	KO.2	1.1	
CDKN1A	K562	2.29	0.004
	KO.1	2.71	
	KO.2	1	
PTPN6_1	K562	2.86	0.021
	KO.1	1.57	
	KO.2	1.57	
VAV1	K562	3	0.001
	KO.1	1.61	
	KO.2	1.39	

Transparent Methods

Cell Culture

K562 cells were maintained in RPMI 1640 media (GIBCO, ThermoFisher Scientific), supplemented with 10% Fetal Bovine Serum (FBS) and 1% penicillin-streptomycin (10,000 U/ml), at 37°C, 5% CO_2. Cells were monitored daily, passaged (split) every ~48 hours and seeded at 2.5 x 10^5 cells in growth media. A hemocytometer was used to observe and count cells for seeding. All cell centrifugations were performed at 1200rpm, for 5 minutes at room temperature unless stated otherwise.

To monitor cell growth, 10^5 cells/ml were seeded into a 6 well-plate and viable cells were counted after 24, 48 and 72 hours. Trypan Blue dye exclusion was used to distinguish viable cells. Before counting, 10ul cell suspension was mixed with 10ul 0.4% Trypan Blue solution (ThermoFisher, Cat: 15250061). 10ul of this mix was then applied to a hemocytometer and cells were counted, excluding those that appeared blue.

Cells were seeded at a density of 2.5 x 10^5 cells/ml and grown in the presence of various inhibitors and drugs. All inhibitors were prepared in a sterile tissue culture hood, and all solvents were filter sterilized before preparing stocks. Control cells were grown in the presence of the equivalent volume of solvent in which each inhibitor was dissolved (E.g. DMSO or H_2O). Upon harvesting after treatment, cells were washed in PBS three times, centrifuging between washes. Cells were grown in growth media containing a final concentration of either 0uM (control), 5uM or 10uM cisplatin (Millipore, Cat: 232120) for 24 hours, before harvesting. A 10mM cisplatin stock was freshly prepared in H_2O for each use. Cells were grown in growth media containing a final concentration of either 0uM (control), 5uM or 10uM 5-Aza-2'-deoxycytidine (DAC) for 48 hours, before harvesting. A 220mM DAC stock was freshly prepared in DMSO for each use.

All cell transfections were similarly performed via electroporation using the Cell Line Nucleofector Kit V (Lonza, Cat: VVCA-1003). For each transfection, 10^6 cells were centrifuged and washed once in 500ul PBS. Stable CCDC26 overexpression (O/x) cell lines were

prepared by first ligating a spliced CCDC26 transcript (isoform 1) into a modified pEF6 plasmid (ThermoFisher #V96220, myc epitope and His-tag were removed previously). Briefly, the resulting plasmid, as well as an empty control pEF6 plasmid lacking CCDC26, were used to transfect both WT and CCDC26 KO cells via electroporation, which were subsequently selected with Blasticidin S HCl (TOKU-E B001). For use in this project, these cell lines were grown in growth media supplemented with a final concentration of 4ug/ml Blasticidin S HCl. For this purpose, a 25mg/ml Blasticidin S HCl stock was prepared in H_2O in sterile conditions and stored at -20°C. CCDC26 overexpression was confirmed via qRT-PCR.

For CRISPR/CAS9 mediated knockouts sgRNA targeting CCDC26 was designed using Wellcome Trust Sanger Institute Genome Editing database (WGE) (Hodgkins *et al*, 2015). sgRNA was selected with minimum off-target effects and close to transcription start site of isoform-1. K562 cells were electroporated with 5μg pX459 vector (Addgene) containing sgRNA targeting CCDC26. Transfected cells were selected for 3 days in a medium containing 0.5 μg/ml puromycin. Selected cells were serially diluted to single cells and were let to grow till colonies were grown. Homozygous mutations were confirmed by amplifying targeted loci using RT–PCR. RT–PCR products were cloned into pJET1.2 blunt vector (ThermoFisher Scientific), and at least 10 bacterial colonies were picked up for genotyping. For CRISPR controls, K562 cells were transfected with pX459 vector without any sgRNAs and cell lines were created similarly as in case of knockouts.

Propidium iodide Fluorescence Activated Cell Sorting (FACS) cell cycle analysis

For cell cycle analysis, ~300,000 cells were centrifuged in FACS tubes. 300ul cold, cell cycle buffer (30μg propidium iodide (PI), 1% (w/v) sodium citrate, 0.1mM $NaCl_2$, 0.1% Triton X-100) was added to the pellet, and gently vortexed, before storing at 4°C for ~24 hours in the dark, to allow PI staining. Depending on the cell cycle stage, cells contain different amounts of DNA. Cells display PI fluorescence that is proportional DNA content and therefore PI fluorescence can be used as a proxy to DNA content. DNA content measurements was performed using a

BD FACS Calibur flow cytometer and data analysis conducted using the BD Cell Quest software (BD Biosciences). For every analysis, 50000 cells were counted with gating to only include single cells. For each sample, extent of propidium iodide staining or DNA content was plotted as a histogram. The cell cycle stages were defined based on DNA content as follows: sub-G1: DNA content < 2n; G1:DNA content = 2n; G2/M:DNA content=4n; Multinucleated cells > 4n. The remaining percentage was attributed to S-phase. The cell cycle boundaries were manually optimized on WT K562 DNA histogram and uniformly applied to all samples as shown in the Figure S1C. The percentage of cells in each cell cycle stage was automatically calculated based on area under curve by BD Cell Quest software. The final values were calculated as mean of three independent experiments.

Cell Fixation

4×10^6 cells were harvested and centrifuged at 1200rpm for 5 minutes at room temperature. Growth media was removed from the pellet, which was then washed in 1ml PBS and centrifuged again as before. The PBS was removed, and the pellet was re-suspended in 50ul PBS. 1ml of a 3:1 mix of Methanol:Acetic Acid fixative was added, gently mixed and incubated at room temperature for 10 minutes exactly. This was followed immediately with centrifugation, followed by three washes with 1ml PBS. Fixed cells were stored in 3ml 70% Ethanol at 4°C at least overnight before use and stored at 4°C for no longer than 3 months.

Immunofluorescence

Approximately 2.5×10^5 fixed cells per slide were aliquoted into sterile microcentrifuge tubes, which were centrifuged for 5 minutes at 7000 rpm, room temperature. The supernatant was removed and the pellet was re-suspended in 200ul PBS. Samples were then spun down onto microscope slides via cyto-centrifugation for 7 minutes at 350rpm.

Cells were blocked by pipetting 50ul of 5% Bovine serum albumin (BSA) (Promega, Cat: W3841) directly onto slides, and incubating for 1 hour at room temperature, followed by a brief

wash in PBS. 50ul of primary antibody (Table S2) diluted in 5% BSA was then pipetted onto each slide and temporarily covered with a 22mm x 22mm coverslip. Slides were placed into a moist chamber and incubated overnight at 4°C. For 5mC immunofluorescence, slides were heated at 94°C for 3 minutes prior to loading primary antibody, in order to separate DNA strands to allow binding.

The following morning, coverslips were removed and slides were carefully washed three times in PBS. Approximately 80ul of the appropriate secondary antibody, diluted in PBS (Table S3) was then pipetted onto slides, sealed temporarily with a coverslip and incubated at room temperature for 1 hour in the dark. This was followed by three PBS washes and a final single wash in H_2O. After allowing slides to dry, 10ul of SlowFade® Gold anti-fade reagent (Invitrogen RNA FISH Kit, Cat. No. F32956) and 1ug/ml DAPI were added to the slide which was then covered with a coverslip and sealed with nail polish.

RNA Fluorescence in situ Hybridization (FISH)

A fluorescently labelled *CCDC26* RNA probe was generated using the FISH Tag RNA Multicolor Kit, Alexa Fluor dye combination (Invitrogen, Cat. No. F32956), following the manufacturers guidelines. A *CCDC26* exon 6 DNA template was generated by performing a PCR using Red Mix (Bioline, Cat. No. BIO25043) WT K562 cDNA and *CCDC26*-specific primers that incorporated a T7 RNA polymerase promoter at the 5'-end of the DNA strand to be later transcribed (Table S1). PCR products ran on a 1% agarose gel and gel purified using the QIAquick Gel Extraction Kit (Cat. No. 28704), following the manufacturers guidelines. The subsequent *CCDC26* DNA template was subsequently used in the first in vitro transcription step of probe generation as directed by the FISH Tag RNA Multicolor Kit.

200ul fixed cells per slide were aliquoted into sterile microcentrifuge tubes, which were centrifuged for 5 minutes at 7000 rpm, room temperature. The supernatant was removed, and the pellet was re-suspended in 500ul Wash Buffer A (5ml 20X nuclease free saline-sodium

citrate (SSC), 5ml deionized formamide, 40ml nuclease-free H_2O). Cells were centrifuged as before and the pellet resuspended in 100ul Hybridization Buffer (1g dextran sulphate, 1ml deionized formamide, 1ml 20X nuclease-free SSC, 8ml nuclease-free H_2O) containing 1ug/ml of probe. This was mixed well by pipetting and incubated overnight, in the dark at 37°C. The following morning, the cells were centrifuged and washed with 500ul Wash Buffer A. Following another centrifugation, the pellet was resuspended in 500ul Wash Buffer A and incubated in the dark, at 37°C for 30 minutes. The cells were centrifuged, and the buffer removed. Cells were then resuspended in 200ul PBS and spun down onto microscope slides via cyto-centrifugation for 7 minutes at 350rpm. 10ul of SlowFade® Gold anti-fade reagent (Invitrogen RNA FISH Kit, Cat. No. F32956) and 1ug/ml DAPI were added to the slide which was then covered with a coverslip and sealed with nail polish.

Confocal Microscopy

Slides were imaged using a Leica TCS SP8 Confocal microscope, using either 20X, 40X or 63X objectives. The brightfield microscope setting was used to visualise cell membrane boundaries. FIJI image analysis software was used to visualise and analyse confocal images.

RNA Extraction

An RNase-free environment was maintained for all RNA work by treatment of equipment and work surfaces with RNase *Zap*, RNase decontamination solution (ThermoFisher Scientific, Cat. No. AM9780).

All RNA was extracted using the QIAGEN RNeasy Mini Kit (Cat: 74106), following manufacturers guidelines. RNA was eluted in 30ul RNase-free H_2O and concentrations were determined using a NanoDrop ND-100 spectrophotometer (Thermo Scientific). RNA quality was determined by running ~200-300ng on a 1% agarose gel.

Nuclear and Cytosolic RNA Extraction

For extraction of nuclear and cytosolic RNA fractions, approximately 10^7 cells were harvested by centrifuging at 1200rpm at room temperature for 5 minutes, washing in 1ml PBS and centrifuging again. As much supernatant as possible was removed from the cell pellet, which was then re-suspended in 1ml of cold (4°C) Buffer RLN (50mM Tris-HCl (pH.8), 1.5mM $MgCl_2$, 140mM NaCl, 0.5% NP-40, 100U/ml RNase inhibitor) and incubated on ice for 5 minutes. 250ul of the mix was then pipetted into four eppendorfs labelled “nuclear”. These were centrifuged for 2 minutes at 3700rpm, 4°C. Approximately 500ul of supernatant collected from all four eppendorfs was collected as cytosolic fraction, taking extra care not to disturb the nuclear pellets. Each of the four nuclear pellets were washed twice with 100ul Buffer RLN, centrifuging for 2 minutes at 3200rpm, 4°C between washes. 200ul and 600ul Buffer RLT (containing 1% β-mercaptoethanol, QIAGEN RNeasy Mini Kit) was added to nuclear and cytosolic fractions respectively and vortexed vigorously. The nuclear pellets were further homogenized using a 1ml syringe and needle (21G x 1.5” – Nr.2., 0.8mm x 40mm). The fractions were then centrifuged for 3 minutes at maximum speed to remove cell debris, and the supernatants were removed and mixed with 1X volume 70% ethanol. The extraction was continued using the QIAGEN RNeasy Mini Kit, following manufacturers guidelines.

All freshly extracted RNA was treated with DNase I using the Sigma, amplification grade DNase I kit (Cat: AMPD1), following the kit guidelines, and converted into cDNA using the Bioline-Tetro cDNA Synthesis Kit (Cat. No. BIO-65042) following the manufacturers guidelines.

Quantitative Real-Time Polymerase Chain Reaction (qRT-PCR)

qRT-PCRs were performed in 96-well plates. 20ng of cDNA made up to 6.8ul with dH_2O was added to each well (in triplicate) with 1.6ul forward primer (5uM), 1.6ul reverse primer (5uM) and 10ul SensiFAST SYBR Hi-ROX mix (Bioline, Cat: BIO92020). The plate was then loaded into an Agilent AriaMax real time PCR machine (Agilent Technologies) and the qRT- PCR was

performed (1 cycle at 95.0°C for 2 minutes, followed by 40 cycles at 95.0°C for 5 seconds, 60.0°C for 10 seconds and 72.0°C for 10 seconds).

The average cycle threshold (Ct) values from each technical triplicate were calculated and used to determine expression levels of the gene of interest relative to the housekeeping gene, GAPDH using the formulae below:

ΔCt = Ct (gene of interest) – Ct (housekeeping gene) $\Delta\Delta$Ct = ΔCt (sample group) - ΔCt (control group)

Relative Expression = $2^{-\Delta\Delta Ct}$

After calculating gene expression relative to the housekeeping gene, GAPDH, unless stated otherwise, values for three biological replicates were averaged and unpaired, two-tailed, parametric *t*-tests were performed to calculate the significance of any differences between control and treated samples. *P* values of <0.05 were considered as statistically significant.

Total Protein Extraction

Total protein was extracted from cells by first harvesting ~2 x 10^6 cells by centrifuging at 1200rpm for 5 minutes. Cell pellets were washed with 1ml PBS and centrifuged again as before. As much PBS as possible was removed from the pellets before re-suspending in ~80ul lysis buffer (20mM Tris, pH 7.5, 150mM NaCl, 10mM EDTA, 0.5% deoxycholic acid, 0.5% Triton X-100). 1 protease inhibitor tablet (Roche, Cat. 04693159001) was added to 10ml lysis buffer immediately before use. Samples were then homogenized using a 1ml syringe and needle (21G x 1.5” – Nr.2., 0.8mm x 40mm) and incubated on ice for 30 minutes before centrifuging at 14,000rpm for 20 minutes at 4°C. The resulting supernatant was pipetted into a fresh, sterile eppendorf and stored at -20°C.

Nuclear and Cytosolic Protein Extraction

Approximately 1.5 x 10^7 cells were harvested by centrifuging at 1200rpm for 5 minutes at room temperature and washed in PBS. As much supernatant was removed from the pellet as

possible, which was then re-suspended in 150ul cold Buffer RLN and incubated on ice for 10 minutes. One protease inhibitor tablet (Roche, Cat. 04693159001) was added to 10ml Buffer RLN immediately before use. The mix was centrifuged at 3700 rpm for 5 minutes at 4°C and 100ul supernatant was transferred to a new eppendorf (cytosolic fraction). The remainder of the supernatant was discarded, and the pellet was washed twice with 100ul Buffer RLN, centrifuging between washes as before. The resulting pellet was re-suspended in ~80ul lysis buffer. One protease inhibitor tablet (Roche, Cat. 04693159001) was added to 10ml lysis buffer immediately before use. Samples were then homogenized using a 1ml syringe and needle (21G x 1.5" – Nr.2., 0.8mm x 40mm) to lyse nuclei, and incubated on ice for 30 minutes before centrifuging at 14,000rpm for 20 minutes at 4°C. The resulting supernatant was pipetted into a fresh, sterile eppendorf (nuclear fraction) and stored at -20°C.

Histone Protein Extraction

10^{7} cells were harvested for histone extraction. Cells were first lysed in 500ul histone extraction buffer (PBS containing 0.5% Triton X-100, 2mM phenylmethylsulfonyl fluoride, 0.02% sodium azide) for 1 minute on ice, followed by centrifuging at 8000rpm for 10 minutes at 4°C. The resulting pellet was washed in 250ul histone extraction buffer and centrifuged as before. The pellet was then resuspended in 0.4M HCl and incubated overnight at 4°C for acid extraction of histones. The following morning, samples were centrifuged as before and the supernatant (containing histone protein) was saved. The HCl was neutralized by adding 0.1X volumes of 2M NaOH. The histone protein was then used in SDS- PAGE (2.7.6). All histone extractions were performed in collaboration with Dr John Halsall (Institute of Cancer and Genomic Sciences, University of Birmingham) (Halsall et al., 2015)

Bradford Assay Protein Quantification

To ensure equal loading of samples in SDS-PAGE, protein concentrations were determined via Bradford assay. Standard samples (5ul of known BSA protein concentration in serial

dilution ranging from 0-2mg/ml) and 5ul of experimental protein samples were loaded in duplicate into a 96-well plate along with 250ul Bradford reagent (Sigma, Cat: B6916). Absorbance was measured at 570nm using a Tecan infinite 5200 pro plate reader and iControl™ Microplate Reader Software. A standard curve was plotted from the standard samples of known protein concentration (conc. vs absorbance). The protein concentration of the experimental samples was determined by subtracting the absorbance from a blank control absorbance value, and then dividing by the slope of the standard curve.

Immunoblotting

For SDS-PAGE, 10% polyacrylamide SDS resolving gels (6.66ml 30% acrylamide, 5ml 1.5M Tris pH8.8, 200ul 10% SDS, 200ul 10% APS, 20ul TEMED and 8ml H_2O) were prepared with a 4% stack (1.7ml 30% acrylamide, 2.5ml 0.5M Tris pH6.8, 100ul 10% SDS, 100ul 10% APS, 20ul TEMED and 5.55ml H_2O) and stored in moist wrapping at 4°C for no more than 1 week before use.

Before loading onto gels, extracted protein samples were mixed with 5X SDS loading dye (200mM Tris-HCL pH6.8, 40% glycerol, 4% SDS, 0.4% bromophenol blue, 200mM β-mercaptoethanol) and heated at 70°C for 10 minutes. In addition to protein samples, 3ul of protein ladder (PageRuler™ Plus Prestained Protein Ladder, ThermoFisher Scientific, Cat: 26619) was loaded onto gels as a molecular weight marker for use as a size standard reference. Gels were then run for approximately 90-120 minutes at 120V in cold 1X Running Buffer (100ml 10X running buffer, 5ml 20% SDS, 895ml dH_2O) (10X running buffer: 60g Tris, 288g glycine, up to 2L dH_2O).

After running, a semi-dry transfer was performed onto nitrocellulose membranes using the Trans-Blot Turbo Transfer System (BIO-RAD, Cat. 1704271) and 1X Transfer Buffer (200ml BIO-RAD TransBlot Turbo 5 x Transfer Buffer (Cat. 1704271), 200ml 100% Ethanol, 600ml nanopure H_2O). Membranes were then blocked in 5% skimmed milk in TBS for 1 hour before incubating in primary antibody overnight at 4°C, with agitation. All primary antibodies (Table S2) were prepared in 5% BSA. The following morning, the membranes were washed three

times in TBS-T for 5 minutes and incubated in the appropriate secondary antibody (Table S3), diluted in blocking solution (5% skimmed milk in TBS-T) in the dark for 1 hour, at room temperature, with agitation. The antibody was removed, and the membranes were washed twice in TBS-T for 5 minutes, and once in TBS for 5 minutes for a final time, before storing in TBS at 4°C.

Membranes were scanned and protein bands were detected using the Odyssey infrared detection system (LI-COR Biosciences). Images were then analyzed and the protein bands were quantified using Image Studio Lite software.

DNA methylation analysis using bisulphite sequencing

For DNA methylation analysis, 1 mg of genomic DNA was sodium bisulphite converted using the EZgold DNA methylation kit (Zymoresearch) following the supplier's protocol and eluted in 50 ml of 1mM Tris-HCl pH 8.0, 0.1mM EDTA. Three ml from this eluate were used for each PCR amplification to prepare the pyrosequencing template, using the PyroMark PCR Kit (Qiagen). PCR volume was 30 ml containing 200 nM of each forward and reverse primer (Table S4). Primers were designed using the Pyromark Assay Design 2.0 software (Qiagen). The thermal profile included an initial denaturation step at 95°C for 15 min, followed by 40 cycles consisted of 94°C for 30 sec, 52°C for 30 sec and 72°C for 30 sec. A final extension step at 72°C for 20 min was utilised. Following quality control of the PCR products on a 2% agarose gel, DNA methylation was measured by Pyrosequencing using the PyroMark Gold Q96 SQA Reagents and a Pyromark Q96 ID instrument (Qiagen), following the manufacturer's protocol. Methylation levels (%) are calculated per CpG site using the Pyromark Q96 2.5.8 software (Qiagen). Methylation index (MtI) represents the mean value of all the CpGs in a target sequence per sample. The difference of DNA methylation between cell lines was tested using the Friedman's non-parametric test, using SPSS 25.0 software.

DNMT1 RNA Immunoprecipitation (RIP)

2 x 10^7 cells were harvested per RIP. Cell pellets were washed in PBS three times and resuspended in 500ul ice-cold Buffer RLN (containing 100U/ml RNase inhibitor and with 1 protease inhibitor tablet added to 10ml RLN). This was incubated on ice for 10 minutes before centrifuging at 3700rpm, for 5 minutes at 4°C. Nuclear pellets were washed with 100ul Buffer RLN, followed by resuspending in 500ul freshly prepared RIP Buffer (25mM Tris, pH 7.4, 5mM EDTA, 150mM KCl, 0.5mM DTT, 0.5% NP40 Igepal, 100U/ml RNase inhibitor) with added protease inhibitor. This mix was incubated on ice for 3 hours with frequent, gentle agitation. Following the incubation, nuclei were homogenized using a 1ml syringe and needle (21G x 1.5" – Nr.2., 0.8mm x 40mm), then centrifuged at maximum speed for 10 minutes at 4°C to pellet nuclear debris. The supernatant was kept and transferred to an RNase- free eppendorf, to which 6ug of either DNMT1 of IgG (control) antibody was added (Table S2). This mix was incubated overnight at 4°C with gentle rotation.

The following day, 50ul (1.5mg) magnetic beads (Dynabeads Protein G Immunoprecipitation kit, Invitrogen, Cat: 10007D) were prepared per RIP. Similar to 2.8, the beads were placed into an eppendorf on a magnetic separator and the supernatant was removed. This was followed by two washes with 100ul RIP buffer. The overnight antibody-protein-RNA suspension was added to the beads and incubated for 1 hour with gentle rotation at 4°C. Following this incubation, the beads- antibody-protein-RNA were placed on the magnet, the supernatant was removed, and the complex was washed three times with 500ul ice-cold RIP buffer and once with RNase-free PBS. At this point, 5% of the bead slurry was collected to be used in SDS-PAGE analysis to confirm DNMT1 pull-down. The remaining beads- antibody-protein-RNA was resuspended in 100ul RIP buffer with 50ug proteinase K and 0.1% SDS. This was incubated at 55°C for 45 minutes to detach the protein- RNA complexes from the beads. The eppendorf was then placed onto the magnet and the supernatant was transferred to a new eppendorf. The beads were discarded.

To purify the RNA, 1X volume of phenol-chloroform-isoamyl alcohol was added to the supernatant and vortexed thoroughly. This was phase separated by centrifuging at 14,000rpm for 10 minutes at 4°C. The aqueous phase (containing the RNA) was carefully collected and placed into a fresh eppendorf. Any remaining aqueous phase was further extracted by the addition of 150ul back extraction buffer (10mM Tris, pH8, 1mM EDTA, 100mM NaCl, 0.25% SDS), followed by vortexing and centrifugation as before. Any remaining aqueous phase was collected and added to the previous collection.

The RNA was further purified by ethanol precipitation. 0.1X volumes of 3M sodium acetate (pH 5.2), 2.2X volumes of 100% ice cold ethanol and 1ul glycogen was added to the RNA extract and incubated at -20°C overnight. The following morning, the mix was centrifuged at 10,000rpm for 20 minutes at 4°C and the supernatant carefully discarded. 500ul 70% ice cold ethanol was added, and the mix centrifuged as before. The ethanol was carefully removed, and the tubes were left open to allow any residual ethanol to evaporate. The purified RNA was then finally dissolved in 20ul RNase-free H_2O. This RNA was then DNase-treated and converted into cDNA, and qRT-PCRs were performed. Results were analysed using the calculations below to determine DNMT1 enrichment for *CCDC26* compared to the IgG control.

DNMT1 RIP ΔCt = Ct DNMT1_RIP_CCDC26 – Ct INPUT_RNA_CCDC26

IgG RIP ΔCt = Ct IgG_RIP_CCDC26 – Ct INPUT_RNA_CCDC26

ΔΔCt = DNMT1 RIP ΔCt – IgG RIP ΔCt Relative Expression = $2^{-\Delta\Delta Ct}$

RNA-sequencing and metagene analyses

RNA-sequencing was carried out on RNA extracted from wild-type K562 cells and CCDC26 KO K562 cells. The sequencing was carried out on three biological triplicates. RNA-sequencing libraries were prepared using TrueSeq method. All the libraries were paired-end sequenced on an Illumina HiSeq 2500 machine (University of Birmingham). Sequences were quality filtered and trimmed using cutadapt. The reads were mapped using TOPHAT package

(Trapnell et al., 2009) against human genome (hg19). Differential analysis was done using cuffdiff programme. Differential genes were identified using false discovery cut-off of 1 x 10^{-5} and used for further analysis. The sequencing data is deposited GEO database (accession no. GSE105029) Metagene plots and heatmap were generated using DeepTools package(Ramirez et al., 2014). DNA methylation metagenes were generated using K562 MeDIP data deposited in ENCODE database (GEO: GSE56774). The GC heatmap were created using previously calculated GC content track for human genome available from UCSC genome browser (http://hgdownload.cse.ucsc.edu/goldenPath/hg19/gc5Base/).

REFERENCES

Halsall, J.A., Turan, N., Wiersma, M., and Turner, B.M. (2015). Cells adapt to the epigenomic disruption caused by histone deacetylase inhibitors through a coordinated, chromatin-mediated transcriptional response. Epigenetics Chromatin *8*, 29.

Ramirez, F., Dundar, F., Diehl, S., Gruning, B.A., and Manke, T. (2014). deepTools: a flexible platform for exploring deep-sequencing data. Nucleic acids research *42*, W187-191.

Trapnell, C., Pachter, L., and Salzberg, S.L. (2009). TopHat: discovering splice junctions with RNA-Seq. Bioinformatics *25*, 1105-1111.
